# Custom Workflow for the Confident Identification of Sulfotyrosine-Containing Peptides and Their Discrimination from Phosphopeptides

**DOI:** 10.1021/acs.jproteome.3c00425

**Published:** 2023-11-08

**Authors:** Leonard A. Daly, Dominic P. Byrne, Simon Perkins, Philip J. Brownridge, Euan McDonnell, Andrew R. Jones, Patrick A. Eyers, Claire E. Eyers

**Affiliations:** † Centre for Proteome Research, Institute of Systems, Molecular & Integrative Biology, University of Liverpool, Crown Street, Liverpool L69 7ZB, U.K.; ‡ Department of Biochemistry, Cell & Systems Biology, Institute of Systems, Molecular & Integrative Biology, University of Liverpool, Crown Street, Liverpool L69 7ZB, U.K.; § Computational Biology Facility, Institute of Systems, Molecular & Integrative Biology, University of Liverpool, Crown Street, Liverpool L69 7ZB, U.K.

**Keywords:** sulfation, phosphorylation, mass spectrometry, fragmentation, neutral loss, PTM, TPST, tyrosyl protein sulfotransferases, heparan-sulfate 6-O-sulfotransferase, secretome

## Abstract

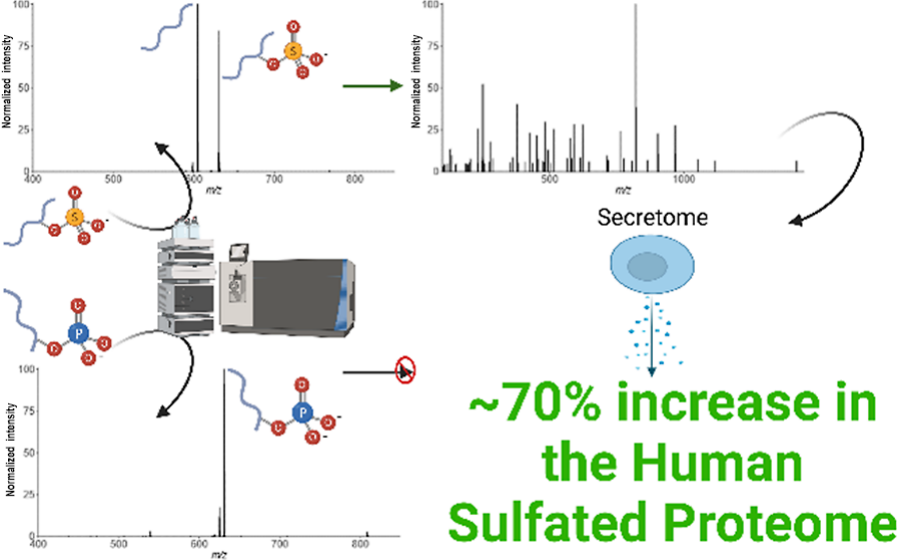

Protein tyrosine sulfation (sY) is a post-translational modification (PTM) catalyzed by Golgi-resident tyrosyl protein sulfo transferases (TPSTs). Information on sY in humans is currently limited to ∼50 proteins, with only a handful having verified sites of sulfation. As such, the contribution of sulfation to the regulation of biological processes remains poorly defined. Mass spectrometry (MS)-based proteomics is the method of choice for PTM analysis but has yet to be applied for systematic investigation of the “sulfome”, primarily due to issues associated with discrimination of sY-containing from phosphotyrosine (pY)-containing peptides. In this study, we developed an MS-based workflow for sY-peptide characterization, incorporating optimized Zr^4+^ immobilized metal-ion affinity chromatography (IMAC) and TiO_2_ enrichment strategies. Extensive characterization of a panel of sY- and pY-peptides using an array of fragmentation regimes (CID, HCD, EThcD, ETciD, UVPD) highlighted differences in the generation of site-determining product ions and allowed us to develop a strategy for differentiating sulfated peptides from nominally isobaric phosphopeptides based on low collision energy-induced neutral loss. Application of our “sulfomics” workflow to a HEK-293 cell extracellular secretome facilitated identification of 21 new sulfotyrosine-containing proteins, several of which we validate enzymatically, and reveals new interplay between enzymes relevant to both protein and glycan sulfation.

## Introduction

Protein phosphorylation involves the reversible, covalent addition of a phosphate group to the side chain of amino acids, predominantly Ser, Thr, and Tyr, alongside 9 nonalcoholic amino acid substrates.^1−3^ As a highly abundant post-translational modification (PTM), phosphorylation has been extensively investigated from both analytical and functional perspectives. It plays critical roles in almost all physiological processes, with an estimated ∼90% of human proteins able to be phosphorylated *in vivo* (the phosphoproteome).^4^ Phosphorylation is catalyzed by protein kinases, and >500 enzymes encoded within the human genome have the ability to catalyze the transfer of the γ-phosphate group from adenosine triphosphate (ATP) to amino acid residues on substrates. Approximately 100 of these kinases are tyrosine-specific,^5^ while others can phosphorylate Ser/Thr and Tyr, or Ser/Thr alone. In contrast, protein sulfation, that is the covalent addition of sulfate which is transferred from the 3′-phosphoadenosine-5′-phosphosulfate (PAPS) cofactor, is poorly characterized analytically and is predicted to be considerably less abundant than phosphorylation. Furthermore, unlike phosphorylation, sulfation is believed to be relatively specific for tyrosine residues, being catalyzed in humans by two separate gene products that encode tyrosyl protein-sulfotransferases (TPSTs), termed TPST1 and TPST2. Some 51 human proteins are annotated in UniProt as containing “sulfotyrosine” (sY), but only 33 of these have been experimentally validated, compared with over 11,000 human “phosphoprotein” entries (accessed May 2023).^6^ Experimental and computational estimates based on site conservation and the biological environment suggest that ∼1–7% of all Tyr residues have the potential to be sulfated (in flies and mice),^7,8^ which potentially makes sY a much more prevalent tyrosine-based PTM than often assumed.

sY was first identified over 50 years ago in fibrinogen/gastrin and appears to be governed (minimally) by acidic consensus motifs in protein targets, which were first characterized biochemically in the early 1980s.^9−12^ Sulfation results in biologically relevant changes in protein/protein affinity, modulating host–pathogen interactions, chemotaxis, FGF7 signaling, proteolytic peptide processing and HIV entry via the viral chemokine co-receptor CCR5.^13−21^ Mice lacking TPST1 have defects in body mass and retinal function, whereas TPST2-deficient mice exhibit hyperthyroidism and infertility.^22^ In contrast, double TPST knockout mice have high perinatal mortality rates due to pulmonary asphyxia and lack detectable sY, confirming that TPST1/2 are together rate-limiting for sY deposition *in vivo*.^22^ While TPST-dependent protein sulfation occurs in Golgi and is believed to be an irreversible modification, sulfation has been most extensively identified on a variety of extracellular/secreted proteins. Interestingly, a recent study reported cytosolic sY of the tumor suppressor p53,^23^ suggesting that the tyrosine “sulfome” may be more extensive and diverse than previously predicted, revealing potential gaps in our current knowledge that are attributable to suboptimal analytical capabilities for high-throughput investigation of sY.

**Figure 1:**
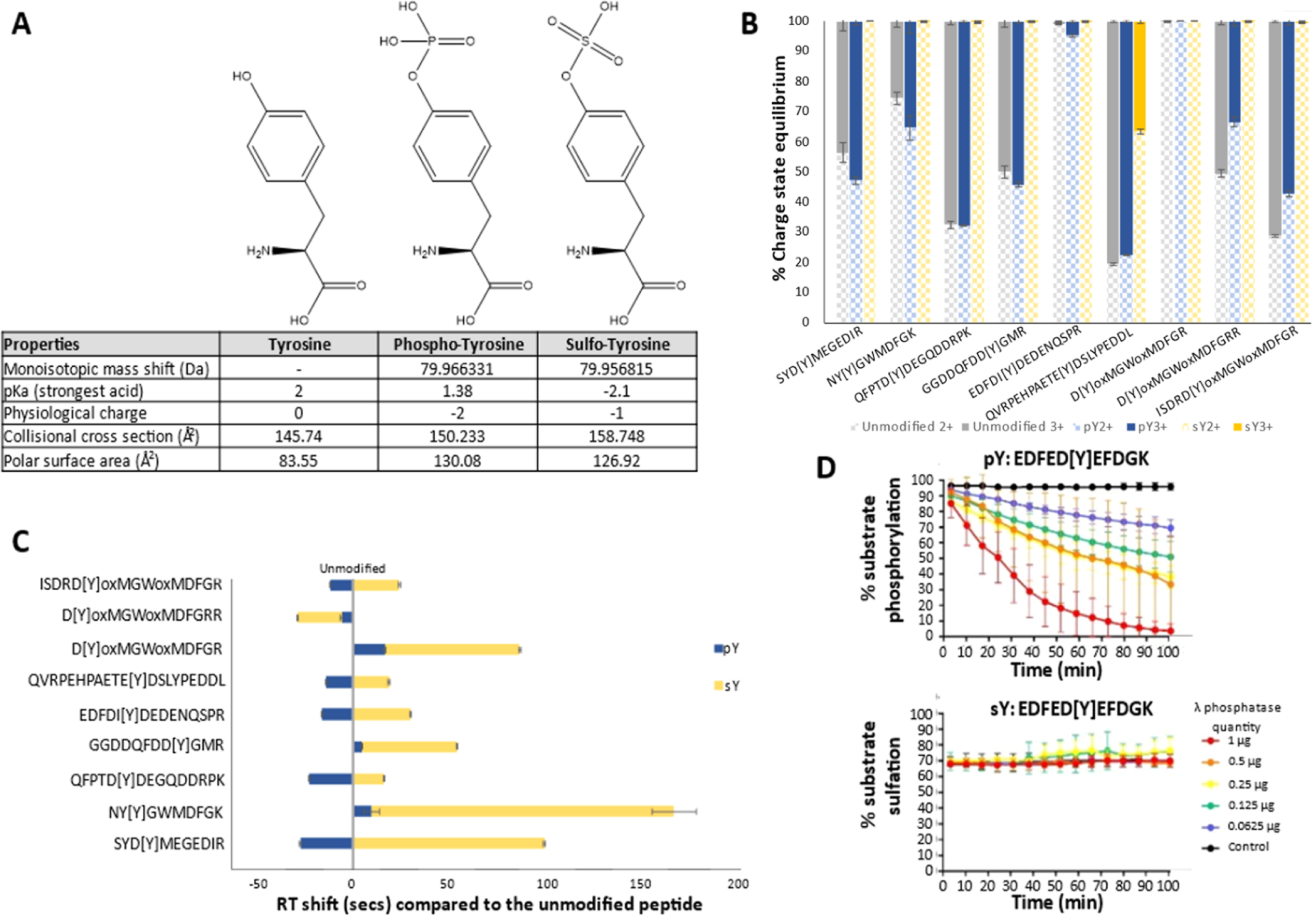
Physiochemical properties of pY vs sY. (A) Chemical structure and properties of tyrosine, phospho-tyrosine, and sulfo-tyrosine. A table of the monoisotopic mass shift (Da) of the phosphate/sulfate moiety in comparison to tyrosine, the p*K*_a_ value of the strongest acidic group, net charge influence at physiological pH (∼8), collisional cross section (Å^2^), and the polar surface area charge-distributed across (Å^2^) are presented. Data were compiled from the 2022 Human Metabolome Database. (B) Charge state distribution of our standard panel of peptides in an unmodified, phosphorylated, or sulfated state. Modification site denoted by [Y], ox = oxidized residue. Error bars represent SE from *N* = 10 replicates. (C) Scaled RT shifts of pY- and sY-peptides. Normalized RT shifts (s) are plotted with reference to the RT of the unmodified peptide counterpart. Modification site denoted by [Y], ox = oxidized residue. Error bars represent SE from *N* = 10 replicates. (D) Real-time λ phosphatase assay to study peptide dephosphorylation or desulfation. Identical fluorescent peptide sequences containing either pY or sY were incubated with stated quantities of λ phosphatase, and the level of modification reversal (dephosphorylation or desulfation) was determined over time (min) using a ratiometric real-time assay. Error bars represent S.D.

Phosphotyrosine (pY) and sY are chemically “similar” with near isobaric mass (sY being 9.6 mDa lighter than pY; pY: 79.966331 Da and sY: 79.956815 Da) and are of comparable size and shape, containing at least one negative charge at physiological pH, formally pY = −2, sY = −1, Figure 1A. Despite these similarities, the best-established phosphopeptide enrichment protocols and gas-phase fragmentation strategies are highly unsuitable for sY-peptide isolation and analysis, stymieing isolation, and mass spectrometry (MS)-based investigations.^24−28^ Currently, sY characterization is reliant on, and highly restricted by, low-throughput TPST screens, for example, on immunoprecipitated putative substrates and/or immunoblotting with monoclonal anti-sY antibodies, ^35^S-radio-labeling, or fluorescent-based enzyme assays.^7,16,19,29−37^ While such techniques are useful for confirming prevalent sY sites in simple mixtures, these approaches are restricted to known or predicted (acidic motif) sites of sulfation, which limits their application for discovery purposes. Hence, there is a clear and pressing need for the development of specifically designed analytical pipelines that are suitable for the global characterization of sulfomes in an untargeted manner.

Recent advancements in MS-based phosphoproteomics pipelines have facilitated the interrogation of phosphorylation-dependent signaling networks to an unprecedented level of detail, consolidating it as an essential technique to explore phosphorylation-mediated signaling. An essential part of these analytical pipelines is the selective enrichment of phosphopeptides based on inherent properties of the appended phosphate moiety, notably a formal net negative charge. Exploitation of standard enrichment strategies, which include titanium dioxide (TiO_2_), anion exchange, and immobilized metal-ion affinity chromatography (IMAC) utilizing various metal ions including Ga^3+^, Ti^4+^, and Fe^3+^, can aid in the analysis of select sY-peptide standards (typically 2–3 non-tryptic, synthetically sulfated peptides). However, these enrichment processes are reported to be highly inefficient.^24−26^ Compounding this, the relatively low ionization efficiency of sY-containing peptides (referred to here as sulfopeptides) and the high lability of the sulfoester bond during MS analysis compromise sensitivity: neutral loss (NL) occurs during collision-induced dissociation (and sometimes during electrospray ionization-MS in the absence of induced fragmentation), generating product ions equivalent to nonmodified fragments, hampering sulfosite localization and discrimination from pY-containing peptides.^27,28,38^

Electron transfer dissociation (ETD) and related hybrid fragmentation strategies such as EThcD and ETciD (which use supplemental collisional dissociation) have proven useful for characterizing peptides that contain labile PTMs, notably phosphorylation.^39−41^ However, sulfopeptides still remain highly prone to sulfate NL.^27^ Direct implementation of electron transfer-mediated approaches in a high-throughput (positive ion mode) proteomics study thus does not appear suitable for sensitive sulfation analysis against the relatively small selection of sY peptides so far investigated.

Halim et al.^42^ report the application of UVPD at 213 nm (as well as infrared multiphoton dissociation and high–low photodissociation) for sulfopeptide characterization. In line with the observations of others,^38,42,43^ they demonstrate reduced stability of a singly protonated sulfopeptide compared with the doubly charged species. While their data indicated that UVPD at 193 nm (and to a lesser degree, 213 nm) provided almost complete sequence coverage for higher charge state sulfopeptide cations, they reported extensive sulfonate loss.

Negative ion mode MS offers potential advantages for sY-peptide characterization, given the acidic nature of both the sY residue and the side chains surrounding the site of modification in most validated substrates, with reports of greater ionization efficiency and less sulfonate NL, akin to that observed for phosphopeptides.^26,38,43^ In particular, negative ETD and negative ion mode electron capture dissociation (niECD) show promise for the analysis and localization of peptide sulfation sites, with niECD generating spectra with complete sulfonate retention on the 5 nontryptic sY-peptides investigated.^43^ Negative ion UVPD also shows promise for localization of sY on peptides, having been used to analyze 5 nontryptic peptides (4 in common with^43^). However, optimal characterization utilizes a custom 193 nm laser that is not currently available on commercial instrumentation.^38^

While there are reports of large-scale negative ion mode proteomics studies,^44,45^ negative ion mode is not routinely applied for HTP studies, in part due to a lack of tested/trained search engines and data analysis tools as well as reduced commercially available options for fragmentation. Computational tools that are developed for automated interpretation of negative ion mode tandem mass spectra will need to take account of the different and more complex fragmentation pathways that arise *cf* positive ion mode during collision-induced dissociation, including multiple, and extensive, side chain NLs.^46−51^

In this paper, we present a discovery proteomics pipeline that can be readily implemented using current commercial instrumentation and search algorithms, that is optimized for the identification of sY-containing peptides from both chemically defined and cellular mixtures, in a manner that permits their discrimination from pY (and other) phosphate-containing peptides. As well as developing a sY-peptide enrichment strategy that employs acetic acid-based solutions with TiO_2_ or IMAC-Zr^4+^, we substantially advance sY-peptide fragmentation studies. In-depth analysis of the largest panel of tryptic sulfopeptides (and phosphopeptide analogues) assembled to date using collision-based, electron-mediated, and UVPD fragmentation strategies allowed us to optimize conditions for sY versus pY discrimination, exploiting the substantive differences in the energy required for sulfonate versus phosphate NL. We also present, and validate, the first application of our sY specific LC–MS/MS-based pipeline for characterization of the sulfome from a complex cellular mixture. The confident identification of sY-proteins includes 21 novel human sulfoproteins, several of which we validate as TPST1/2 substrates using *in vitro* sulfotransferase assays and MS-based tryptic peptide characterization.

## Experimental Section

### Reagents

Powdered chemical reagents were purchased from Merck. Custom peptides (unmodified and phosphorylated) were purchased from Thermo Fisher as PEPOTEC light grade 2 purity. High-performance liquid chromatography (HPLC)-grade solvents for MS were purchased from Thermo Fisher Scientific. All Eppendorf tubes used are ultrahigh recovery Eppendorf tubes (STARLAB). PAPS (adenosine 3′-phosphate 5′-phosphosulfate, lithium salt hydrate) was purchased from Merck and stored at −80 °C to afford maximal stability.

### Purification of Recombinant Proteins

Human TPST1 (residues Lys43–Leu360) and TPST2 (residues Gly43–Leu359) (both lacking the transmembrane domains) were purified as recombinant proteins with an N-terminal 6xHis-tag and a 3C protease cleavage site using pOPINF (OPPF-UK) and refolded *in vitro* into a catalytically active conformation, as previously described.^30^ Lambda (λ) protein phosphatase was purified to homogeneity as previously described.^52^ The tyrosine kinase EphA3, which autophosphorylates on Tyr when expressed in *Escherichia coli*, comprised the kinase domain and the juxtamembrane region with an N-terminal 6xHis-tag, was expressed in pLysS *E. coli* from the pET28a LIC vector, and purified using Ni-NTA agarose and size exclusion chromatography.^53^

### *In Vitro* Sulfation of Peptide Standards

Peptide sulfation assays were performed as described previously.^30^ Briefly, 2.5 μg of each peptide in individual reactions was incubated at 20 °C for 18 h in the presence of 1 μg of TPST1 and TPST2 and 0.5 mM of the cofactor PAPS, the source of the transferred sulfate. *In vitro* sulfated peptides were combined before undergoing stage-tip-based strong cation exchange cleanup.^54^ The eluent was aliquoted into 0.25 μg/peptide fractions and vacuum-dried by centrifugation prior to analysis.

### Protein Phosphatase Treatment

For cell-based experiments, a final concentration of 2 mM MnCl_2_ and 1 mM dithiothreitol was added to the sample followed by addition of a 100:1 (w/w) ratio of peptide: purified λ phosphatase. Samples were incubated at 37 °C for 2 h with constant shaking at 600 rpm.

### Enzymatic sTyr and pTyr Peptide Modification Assays

A 5-FAM fluorophore (maximal excitation of 495 nm/maximal emission of 520 nm, detectable by a LED-induced fluorescence) was covalently coupled to the N-terminus of a custom peptide substrate containing a single modifiable Y site embedded in a canonical acidic motif (5-FAM-EDFED[**Y**]EFDG-CO-NH_2_), which was purchased from Pepceuticals (Leicester, U.K). The peptide was enzymatically Tyr sulfated as described above, or enzymatically Tyr phosphorylated using the same buffer conditions, but substituting TPST1 and 0.5 mM PAPS with the tyrosine kinase EphA3 and 5 mM Mg/ATP. The PerkinElmer LabChip EZ II Reader system, 12-sipper chip and CR8 coating, and assay separation buffer, were all purchased from PerkinElmer and employed as reported previously.^55,56^ Pressure and voltage settings were adjusted manually to afford optimal separation of modified (sY and pY) vs unmodified peptides. Individual phosphatase assays were performed (in triplicate) in a 384-well plate in a volume of 80 μL using 2 μM peptide in the presence of the indicated quantity (μg) of λ phosphatase, 50 mM HEPES (pH 7.4), 0.015% (v/v) Brij-35, 5 mM MnCl_2_, and 1 mM DTT. The degree of peptide sulfation/phosphorylation was directly calculated by the EZ Reader software by differentiating modified/unmodified phosphorylated or sulfated peptide peak ratios.

### Titanium Dioxide Enrichment

Dried peptide standards were resuspended in the appropriate solution to a concentration of 0.2 μg/μL by water bath sonication for 10 min. TiO_2_ resin (GL-Sciences) was added to a final amount of 5:1 (w/w) TiO_2_ resin/peptide and incubated at 25 °C with 1500 rpm shaking for 30 min before centrifugation at 2000*g* for 1 min at room temperature and the supernatant removed. TiO_2_ resin–peptide complexes were washed 3× for 10 min with 1500 rpm shaking in the same solution, resuspending in an equal volume after centrifugation as before. TiO_2_ resin was vacuum centrifuged for 15 min, prior to addition of an equal volume of 5% ammonium hydroxide (in water) and shaking at 1500 rpm for 10 min. Samples were centrifuged as before, and the supernatant was collected and dried to completion under vacuum centrifugation.

### Labeling NTA-Agarose-Coated Magnetic Beads

PureCube NTA MagBeads (Cube BioTech) were labeled according to the manufacturer’s protocol, scaling to 100 μL of resin slurry using magnetic support racks. Prelabeled Ti^4+^ and Zr^4+^ PureCube MagBeads were washed following the labeling protocol. All beads were made in a 1% (w/v) stock and stored at 4 °C prior to use.

### IMAC Enrichment

An adapted version of the TiO_2_ enrichment protocol described above was used. Alterations include using a 2.5:1 (w/w) ratio of IMAC resin/peptide and washing the required quantity of beads twice in resuspension solution using a volume 5-fold that of the bead slurry prior to the addition to peptides.

### C18 Stage-Tip Cleanup

sY-peptides for fragmentation studies, standard input samples, and spiked into BSA/Casein digests were subjected to in-house C18 stage-tip (Empore Supelco 47 mm C18 extraction discs) cleanup prior to LC–MS/MS analysis. Three discs were packed per 200 μL tip and centrifuged at 5000*g* for 5 min to pack the column. All centrifugation steps were at 4000*g* for 2 min (or until all liquid had passed through the tip) at room temperature. Stage-tips were equilibrated by sequential washing with 200 μL of methanol, elution solution (80% (v/v) ACN (acetonitrile) + 0.1% (v/v) TFA in water) and wash solution (0.1% (v/v) TFA in water) prior to loading peptide samples in 0.1% (v/v) TFA. Flow through was reapplied before washing in 200 μL of wash solution and eluting in 200 μL of elution solution. Elution was dried to completion by vacuum centrifugation.

### Liquid Chromatography–Tandem Mass Spectrometry Analysis

All dried peptides were resuspended in 3% (v/v) ACN + 0.1% TFA (in water) by water bath sonication for 10 min, followed by centrifugation at 13,000*g* at 4 °C for 10 min, and the cleared supernatant collected. For protein/peptide standards, peptides were separated by reversed-phase HPLC over a 30 min gradient using an Ultimate 3000 nano system (Dionex), as described in.^57^ All data acquisition was performed using a Thermo Orbitrap Fusion Lumos Tribrid mass spectrometer (Thermo Scientific) over a *m*/*z* range of 350–2000. MS1 spectra were acquired in the Orbitrap [120 K resolution at 200 *m*/*z*], normalized automatic gain control (AGC) = 50%, maximum injection time = 50 ms, and an intensity threshold for fragmentation = 2.5 × 10^4^. MS2 spectra were acquired in the Orbitrap [30k resolution at 200 *m*/*z*], AGC target = normal and maximum injection time = dynamic. A dynamic exclusion window of 10 s was applied at a 10 ppm mass tolerance. For cell-based studies, peptides were separated and acquired with either of the following adaptions: (1) a 60 min gradient and higher-energy C-trap dissociation (HCD) set at 32% normalized collision energy (NCE). (2) For NL triggered methods, HCD set at 10% NCE, MS2 acquired in the Orbitrap [15k resolution at 200 *m*/*z*] and targeted loss continuation trigger for *m*/*z* values equivalent to 1–3 sites of sulfation (79.9568 amu) at charge states +2 to +5 (25 ppm mass tolerance). Continuation of the trigger was performed for NL ions with at least 10% relative intensity for correct charge state-assigned losses. Triggered scans were acquired in the Orbitrap [30k resolution at 200 *m*/*z*], HCD set to 32% NCE, AGC target = standard and maximum injection time = auto.

### MS Data Analysis

The panel of synthetic peptide standards and the products of immunoprecipitated heparan-sulfate 6-*O*-sulfotransferase 1/2/3 (H6ST-1/2/3) *in vitro* Tyr sulfation assays were analyzed using Proteome Discoverer 2.4 (Thermo Scientific) in conjunction with MASCOT^58^ against either a custom database of all (12) peptides, BSA and Casein (αS1, αS2, β) isoforms only, or UniProt Human Reviewed database [updated May 2023] for the H6ST immunoprecipitation experiments. Data were imported into Skyline for calculating charge state distributions and retention time (RT) shifts based off *m*/*z* values from Proteome Discoverer 2.4 analysis. Secretome data were converted into MZML format using MSConvert^59^ with peak picking “2-” filter applied. For NL data sets, an additional filtering parameter of “HCD energy 32” was applied. MZML datafiles were searched using PEAKS11 against the UniProt Human reviewed database (updated June 2022). All data were searched using trypsin (K/R, unless followed by P) with 2 miscleaves permitted and constant modifications = carbamidomethylation (C), variable modification = oxidation (M), sulfation (STY), and phosphorylation (STY). NL-triggered methods were additionally searched using semispecific trypsin and the additional variable modification of deamidation (NQ). MS1 mass tolerance = 10 ppm, MS2 mass tolerance = 0.01 Da, instrument type = Orbitrap (Orbi–Orbi), fragmentation = HCD, data-dependent acquisition = DDA. All data were filtered to 1% FDR.

### Informatics and Localization Data Analysis

For localization data analysis and heatmap preparation, synthetic peptide MS data sets were searched in Comet (with parameters matched to the PEAKS11 search), which enables export of all peptide spectral matches (PSMs), without any machine-learning-based rescoring (as in PEAKS11) or score thresholding. Heat maps were produced in R (tidyverse, ggplot2) for every fragmentation mode (for phosphopeptides and sulfopeptides at multiple charge states). The heat map displays, for correctly identified PSMs, the proportion of the identified count of fragment ions containing the known modification site divided by the total possible count of theoretical observable ions containing the known modification site.

### Cell Culture, Secretome Precipitation, and Sample Preparation

Adherent HEK-293 cells were seeded at a density of ∼1.75 × 10^5^ cells/cm^2^ in DMEM supplemented with 10% (v/v) fetal calf serum, 1% (v/v) nonessential amino acids, and 1% (v/v) penicillin/streptomycin and maintained at 37 °C, 5% CO_2_ until ∼80% confluent (∼2 × 10^8^ cells). Cells were washed 2× in 10 mL of phosphate buffered saline (PBS) before incubation for 18 h in serum-free growth medium. The HEK-293 “secretome” was collected from the medium and centrifuged at 200*g* for 5 min and then 10,000*g* for 5 min, preserving the cleared supernatant each time. Secreted proteins were captured by addition of 100:1 (v/v) secretome/strataclean resin (Agilent Technologies) (∼2 mL) and incubated at room temperature with end over end rotation for 2 h. Strataclean resin–protein complexes were centrifuged at 500*g* for 2 min and the supernatant removed. Complexes were washed twice in an equal volume (relative to initial secretome) of PBS, resuspended in 1 mL of PBS, transferred to a 1.5 mL centrifuge tube, and washed in (3×) 1 mL of PBS. Proteins were eluted in 1 mL of 5% (w/v) SDS, 500 mM NaCl, and 50 mM Tris (pH 8) at 80 °C for 10 min, with brief vortexing every 2 min, followed by centrifugation at 13,000*g* for 10 min at room temperature. Protein concentration was determined by the BCA assay before samples were reduced and alkylated with dithiothreitol and iodoacetamide.^57^ A 1:1 mixture of magnetic hydrophobic and hydrophilic Seramag speedbeads (MERCK) was added at a 2:1 (w/w) ratio of beads/protein and ACN was added to a final concentration of 80% (v/v) and incubated at 25 °C with shaking (1500 rpm) for 30 min. The supernatant was discarded, and beads were washed (3×) in 200 μL of 100% ACN. Beads were dried by vacuum centrifugation for 10 min and resuspended by water bath sonication for 2 min in 1 mL of 100 mM ammonium acetate, pH 8. Trypsin Gold (Promega) was added at a 33:1 (w/w) protein/trypsin ratio and incubated overnight at 37 °C with 1500 rpm shaking. The peptide containing the supernatant was transferred into a fresh tube, and the remaining beads were then washed with a 2× volume (cf to bead volume added) of 8 M urea in 100 mM ammonium acetate, pH 8, for 30 min with 1500 rpm shaking before the supernatants were combined. The pooled sample was incubated on ice for 30 min and centrifuged at 13,000*g* for 10 min at 4 °C and the cleared supernatant collected. Samples were λ phosphatase-treated (as described) prior to acidification with a final concentration of 0.5% (v/v) TFA and incubated at 37 °C for 30 min followed by 4 °C incubation for 30 min. Samples were centrifuged at 13,000*g* for 10 min at 4 °C and the cleared supernatants collected. Samples were split 1:33:33:33, respectively, for total protein, agarose coated IMAC-Zr^4+^, BioResyn IMAC-Zr^4+^ HP, or TiO_2_ sY enrichment protocols prior to drying to completion by vacuum centrifugation.

### Functional Enrichment Analysis

DAVID Bioinformatics Resources [v6.8]^60^ was used to determine the cellular compartments (and biological processes) in the secretome-enriched protein sample.

### Heparan-Sulfate 6-*O*-Sulfotransferase 1/2 Immunoprecipitation and *In Vitro* Sulfation Assay

The cytoplasmic regions of human HS6ST1 (37–410), HS6ST2 (228–605), and HS6ST3 (28–471) were cloned into pcDNA3 with a 3C-protease cleavable, N-terminal tandem StrepTag for human cell expression. HEK-293T cells were transfected at ∼40% confluency using a 3:1 polyethylenimine (branched, average *M*_w_ ∼25,000 Da; Merck) to DNA ratio (30:10 μg, for a single 10 cm culture dish). Cells (from 10 × 10 cm culture dish) were pooled and harvested 48 h post transfection in lysis buffer (150 μL per dish) containing 50 mM tris–HCl (pH 7.4), 150 mM NaCl, 0.5% (v/v) Triton X-100, 2 mM CaCl_2_, 10 mM MgCl_2_, and 20% (v/v) glycerol and supplemented with protease and phosphatase inhibitors (Roche). Lysates were sonicated briefly on ice and clarified by centrifugation at 20,817*g* for 20 min at 4 °C, and the resulting supernatants were incubated with 15 μL of Strep-Tactin sepharose resin (Thermo Fisher Scientific) for 3 h with gentle end over end mixing at 4 °C. Sepharose beads containing bound protein were collected and washed three times in 50 mM Tris–HCl (pH 7.4) and 500 mM NaCl and then equilibrated in storage buffer (50 mM Tris-HCl (pH 7.4), 100 mM NaCl, and 5% (v/v) glycerol). HS6STs were then proteolytically eluted from the beads over a 2 h period using 3C protease (0.5 μg) at 4 °C with gentle agitation. Purified HS6STs (10 μL of the 35 μL elution volume) were sulfated with TPST1, TPST2, or both TPST1 and 2 (as described above) for 18 h at 20 °C.

## Results and Discussion

### sY and pY Have Distinct Biochemical and Analytical Properties

While the masses of sY and pY moieties are nominally isobaric, they possess distinct chemical properties (Figure 1A;^61^). At physiological pH (∼7.4), sY carries a single net charge of −1, while pY has a net charge of −2. The primary (most acidic) p*K*_a_ of sY is −2.1 compared with 1.38 for pY (as reported in the Human Metabolome database) (;^62^http://www.hmdb.ca). Based on molecular dynamics simulations and experimental analysis, sY exhibits a marked reduction in the potential for hydrogen bonding interactions compared with pY.^61^ This difference in electronegativity likely explains the previously reported reduced ionization efficiency of sY-peptides in positive ion mode and the lability of the sulfonate group during proton-driven collision-induced dissociation.^27,28,38^

To investigate how sY or pY affects peptide analysis, we synthesized a panel of 12 Tyr-containing peptides designed on the basis of known/putative sulfation sites from proteins. We also generated an analogous panel containing pY. The peptides were enzymatically sulfated using TPST1 and 2^30^ to generate a panel of tryptic sY-peptides for comparison against synthetically generated phosphopeptides modified on the same residue (Table S1 and Figure 1A). All peptide variants (either unmodified, pY- or sY-containing) were analyzed under standard positive ion mode LC–MS/MS conditions, comparing charge states and RTs (Figure 1B,C). Only peptides identified in all forms (unmodified, pY and sY) across 10 replicate analyses were included (a total of 9 peptides). We identified little difference in the relative abundance of the +2 and +3 charge states for peptides when comparing the unmodified and pY forms (Figure 1B). In marked contrast, and consistent with previous studies,^38,63^ we observed a significant reduction in the detection of +3 sY-peptide ions, preferentially observing doubly protonated species; only 1 out of the 9 peptides in this set (QVRPEHPAETE[sY]DSLYPEDDL) presented in its [M + 3H]^3+^ form (likely due to the presence of internal Arg and His residues) with all others appearing almost exclusively as +2 species. Singly protonated sY peptide ions, when present, were at substantially lower levels (<1% relative intensity compared to +2 ions).^38,42,43^ Comparing LC RTs for sY-peptides versus their unmodified counterparts over the rapid 10 min LC gradient, we observe a clear increase (ranging from 16 to 156 s, that is, a RT shift of between 2.0 and 17.6%) for 8 out of the 9 sY-peptides. Conversely, and as previously reported, pY-peptides exhibited faster LC elution times (ranging from 5 to 26 s) (Figure 1C).^64,65^ Interestingly, D[Y]oxMGWoxMDFGRR was the only peptide where RT decreased due to sulfation and indeed eluted earlier than the pY variant (−22 s for sY vs −5 s for pY). This peptide has the highest pI value of peptides in this panel (pI = 5.96, range = 3.62–5.83, Table S1) and contains two Arg residues, suggesting that there is no direct relationship between pI and modification type, and hydrophobicity.

Given the comparatively high prevalence of phosphorylated proteins compared to known sulfoproteins in the human proteome, we explored whether enzymatic phosphatase pretreatment could reduce the relative abundance of phosphopeptides and thereby (i) minimize possible mis-identification of sY-peptides, particularly given the very small mass shift between the two moieties (∼9.6 mDa), and (ii) improve the sensitivity of sulfoproteomics pipelines by minimizing coenrichment of sulfopeptides and phosphopeptides. Protein phosphatase treatment combined with MS has previously been used to investigate sY-peptide identification, discriminating from pY due to a lack of activity and thus an absence of mass shift post treatment.^66,67^ We confirmed the specificity for dephosphorylation by monitoring activity toward pY- and sY-forms of the same fluorescently labeled peptide substrate (EDFED[Y]EFDGK) using a phosphorylation assay^30^ in reverse (Figure 1D) similar to the procedure we developed for real-time desulfation analysis of glycans.^68^ Irrespective of the quantity of protein phosphatase employed (μg) and under conditions with essentially complete dephosphorylation of the pY-peptide (>95%), no loss of the sY-containing peptide was observed, confirming that protein phosphatase pretreatment is likely an effective means of reducing phosphopeptide content in biological samples, while preserving peptide sulfation prior to enrichment and MS analysis.

### Acetic Acid-Based Solutions with TiO_2_ or Zirconium^4+^ IMAC Can Efficiently (and Semi-preferentially) Enrich sY-peptides

MS-based characterization of PTMs in complex mixtures requires enrichment of peptides (or proteins) to improve detection sensitivity and overcome the fact that PTM-modified peptides usually represent a small proportion of the total peptide pool.^1,69−71^ The selectivity of phosphate-enrichment techniques is based on PTM-specific biochemical properties or antibodies. Low pH IMAC and TiO_2_ have thus become staples in phosphoproteomics pipelines.^72−74^ Both TiO_2_- and IMAC-based phosphopeptide enrichment strategies exploit the relatively low p*K*_a_ of the phosphate group (the primary p*K*_a_ value of phosphate monoesters being ∼2) compared with the side chains of other amino acids (the most comparable being Asp and Glu with p*K*_a_ values of ∼3.5 and 4.2 respectively^75^) to promote efficient and (relatively) specific binding. By maintaining a low pH, it is therefore possible to preferentially enrich phosphopeptides, as opposed to Asp/Glu-rich peptides with positively charged immobilized metal ions.

However, application of these approaches for the enrichment of sY-peptides is potentially complicated by two major factors; first, it is reported that sY undergoes acid-induced hydrolysis,^76,77^ which would reduce the recovery of sY using standard low pH enrichment conditions. However, we failed to detect acid-induced sulfate hydrolysis across our peptide panel, even after sample storage for a week at 4 °C in 0.1% (v/v) TFA (data not shown), in line with some other reports.^26^ Second, the single hydroxyl group of sY and the size and orientation of the sulfonyl group (Figure 1A) might prohibit the efficient capture of sY-peptides by TiO_2_. Phosphopeptide enrichment with TiO_2_ relies on the spatial coordination and bidentate hydrogen bonding of the phosphate group to resin-chelated titanium ions;^78−80^ the reduced hydrogen bonding capacity of sY-peptides may compromise TiO_2_ binding. Indeed, a previous report that used a commercial TiO_2_ kit reported no enrichment of two sY-peptides.^26^ However, the binding and wash solutions used in this kit are proprietary, and mobile phase composition is known to play a significant role in the efficiency of such enrichment processes.^81,82^ Moreover, although sTyr antibodies exist, these are unreliable in our hands for immunoprecipitating proteins or peptides containing sTyr.

**Figure 2:**
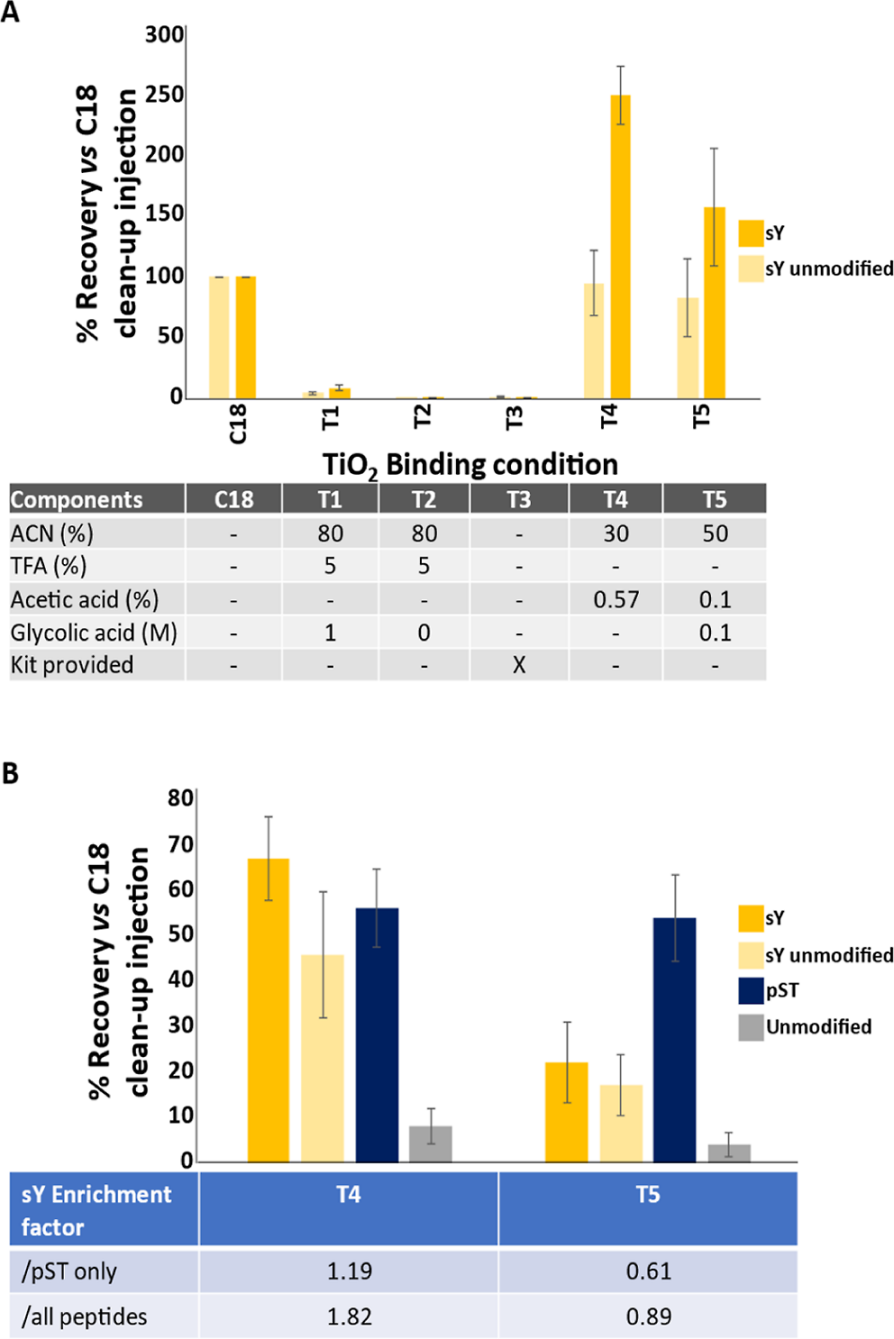
TiO_2_-based enrichment of sY-peptides. (A) Solution optimization for recovery of sY-peptides. The panel of sY- and unmodified peptides was enriched using standard TiO_2_ protocols with different loading and wash solutions as stated and analyzed by LC–MS. Recovery was determined by normalizing to an equal amount of “unenriched” input material subjected to C18 reverse phase cleanup. Enriched samples were not subject to C18 cleanup. Recovery of the 8 sY- and 9 unmodified peptides is an average (mean ± S.D.) of all equivalent (non)modified peptides, including methionine oxidized variants which we have previously shown, has little effect on relative signal intensity.^87^ (B) A mixture of trypsin digested BSA, casein-αS1, -αS2 and -β and the sY-peptide panel was subjected to TiO_2_ enrichment using T4 and T5 as specified in (A) and efficiency of enrichment of sY, pS/T, and unmodified peptides determined. All samples were subjected to C18 cleanup prior to LC–MS/MS analysis. Recovery (mean ± S.D.) (compared with “unenriched” input material subjected to C18 reverse phase cleanup) was determined for 8 sY-, 9 unmodified synthetic peptides, as well as 9 pST peptides from casein and 19 unmodified peptides from BSA and casein.

**Figure 3:**
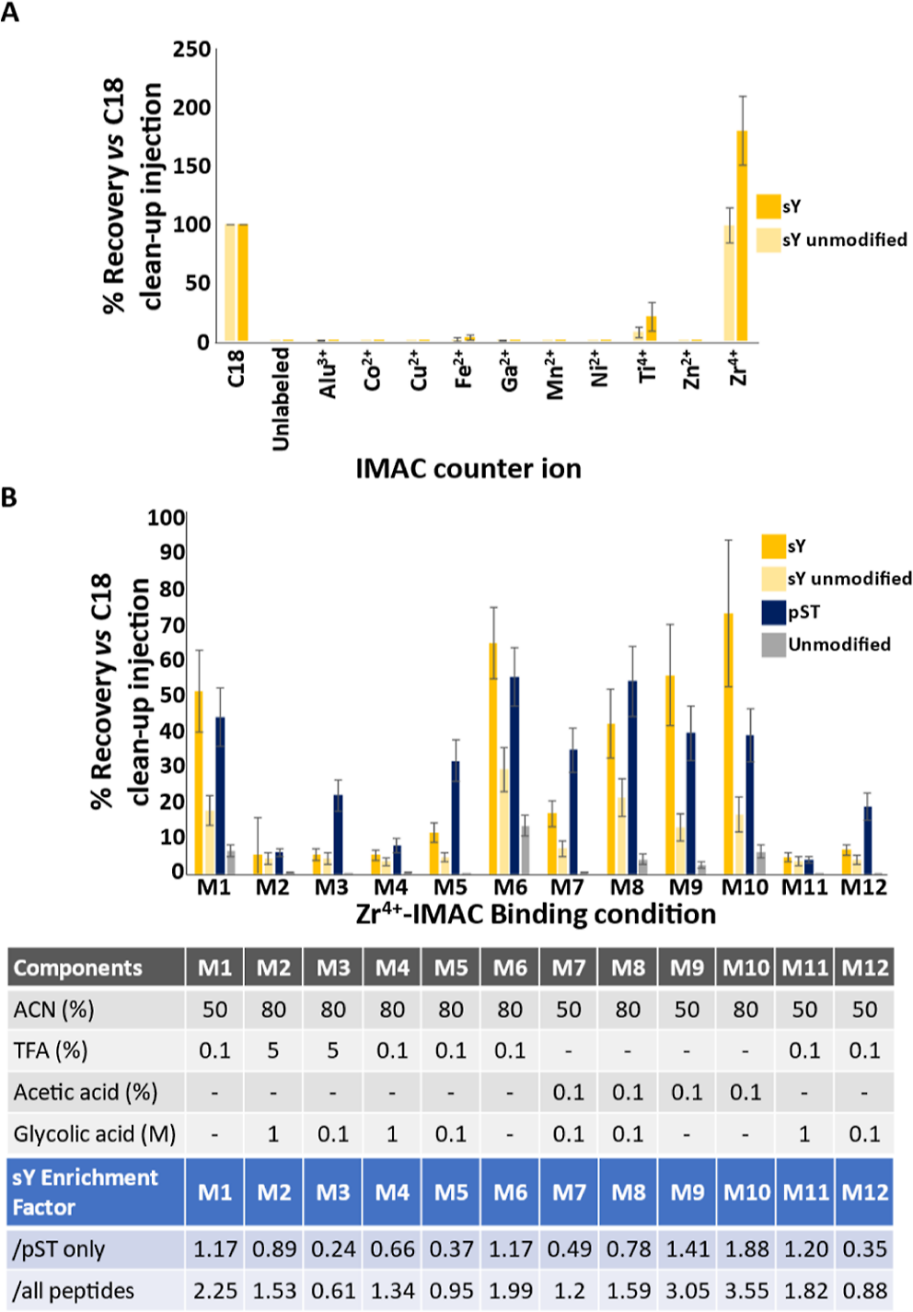
Development of an IMAC-based enrichment protocol for sY-peptides. (A) Efficiency of recovery of the panel of sY- and unmodified peptides was evaluated for 10 IMAC counterions as indicated. Recovery was determined by normalizing to an equal injection of “unenriched” input material subjected to C18 reversed phase clean up. Recovery of the 8 sY- and 9 unmodified peptides is an average (mean ± S.D.) of all equivalent (non)modified peptides, including methionine oxidized variants, which we have previously shown has little effect on relative signal intensity.^87^ (B) Optimization of Zr^4+^ recovery of sY-peptides. A mixture of trypsin digested BSA, casein-αS1/αS2/β, and the sY-peptide panel were subjected to enrichment with Zr^4+^ IMAC using different loading solutions as indicated. All samples were subjected to C18 cleanup prior to LC–MS/MS analysis. Recovery (mean ± S.D.) was determined for 8 sY-, 9 unmodified synthetic peptides, 9 pST peptides from casein, and 19 unmodified peptides from BSA and casein.

While Fe^3+^-IMAC^26^ and Ga^3+^-IMAC^25^ have been tested for the enrichment of sY-peptides, the relative efficiency has not been carefully evaluated. Other metal counterions have also proven useful for phosphopeptide enrichment.^83−85^ However, there has not been a comprehensive evaluation of the utility of different IMAC counterions for sY-peptide enrichment. Previous attempts at sY peptide enrichment did not consider tryptic peptides or fully explore the efficiency or selectivity of sY peptide enrichment as a function of binding and wash conditions. Given our desire to advance sTyr analysis in a variety of biological mixtures, we undertook a comprehensive quantitative evaluation of the ability of different immobilized media, including TiO_2_ and IMAC (comparing 10 different metal counterions), in terms of specificity and efficiency for enriching tryptic sY-peptides (Figure 2 and 3).

Initial evaluations focused on TiO_2_-based enrichment using solutions typically employed for phosphopeptides and sample loading conditions previously shown to enable binding of three (nontryptic) sY-peptides^86^ (Figure 2). To rule out nonspecific enrichment resulting from the increased hydrophobicity of the sY modification, sY peptide recovery rates for each condition were directly compared to the amount loaded following C18 reverse phase chromatography as a “cleanup” step. Under standard TiO_2_ phosphopeptide enrichment conditions (80% ACN, 5% TFA, 1 M glycolic acid, T1^57^), application of a modified loading solution without glycolic acid (80% ACN, 5% TFA, T2) or using the commercially available Phos-TiO_2_ spin-tip kit (GL Sciences, T3), we failed to observe efficient recovery or enrichment (<5%) of any sY-containing peptides (Figure 2A). Little to no recovery was observed for nonmodified peptides as would be expected. Reducing the acetonitrile content and/or replacing TFA with lower concentrations of acetic acid (30% ACN, 100 mM acetic acid, T4; 50% ACN, 0.1% acetic acid, 0.1 M glycolic acid, T5) improved recovery of all peptides, with ∼2.5-fold and 1.5-fold enrichment of sY-peptides for conditions T4 and T5, respectively, compared with the C18-desalted control (Figure 2A). Recovery of sulfated peptides was substantially greater (>100%) following enrichment in the presence of acetic acid than for peptides subjected to C18 cleanup (as used for normalization of recovery), indicating that this additional C18 step compromises sulfopeptide recovery and should ideally not be used in sulfomics workflows.^87^

To evaluate sY-enrichment in a mixture of tryptic synthetic sY peptides alongside phosphopeptides derived from purified BSA and α/β casein (at molar excesses of 170× and 90× respectively), we compared the relative enrichment of sY- versus pST-peptides in these samples. From this peptide mixture, we quantified recovery of sY peptides, unmodified peptides (lacking sTyr but still highly acidic), 9 phosphopeptides from casein, and 19 unmodified peptides (10 from BSA and 9 from casein). Signal intensity was normalized to an equal load of non-TiO_2_-enriched material, all samples having been subjected to C18 cleanup (Figure 2B). The sY enrichment factor was then determined by comparing the relative recovery of sY-peptides with respect to either all peptides observed or phosphopeptides only.

As previously observed for the synthetic peptides (Figure 2A), sY peptide recovery from this mixture was much lower with the T5 condition than T4, while there was little variation (±<5%) in the relative recovery of either phosphorylated or nonphosphorylated peptides from BSA/casein. Consequently, a greater enrichment factor was observed for T4 than T5, whether normalized against all peptides (1.82 or 0.89 respectively) or just the phosphopeptide cohorts (1.19 or 0.61 respectively). While these results may be explained in part by the reduced acetonitrile content of T4, we hypothesize that the glycolic acid may be acting to reduce nonspecific binding of the acidic residues in the sY peptides given the comparative recovery of the unmodified synthetic peptides. Thus, we were able to partially enrich for sY-peptides and believe that this is primarily due to the high acidic content of TPST1/2-consensus containing peptides.

We next investigated sY-peptide enrichment using IMAC and a panel of 10 metal counterions (Al^3+^, Co^2+^, Cu^2+^, Fe^2+^, Ga^3+^, Mn^2+^, Ni^2+^, Ti^4+^, Zn^2+^, and Zr^4+^). Under relatively mild conditions (50% ACN, 0.1% TFA), Zr^4+^ exhibited by far the most efficient recovery (∼180% cf. C18 enrichment) across all sulfopeptide standards, with Ti^4+^ being the only other counterion capable of enriching multiple sulfopeptides, albeit rather inefficiently, with a recovery of ∼20% (Figure 3A). In contrast with previously published data which used Ga^3+^ and Fe^3+^,^25,26^ we did not observe sulfopeptide capture using Ga^3+^-IMAC and only minimal recovery (∼3%) was seen with Fe^3+^-IMAC.

Given the comparatively high recovery of sY peptides with Zr^4+^-IMAC, we evaluated the effect of different binding conditions on the recovery and enrichment of sY peptides in a more complex BSA/casein/synthetic peptides mixture, as performed above for TiO_2_. Overall, we evaluated 12 different conditions, altering the concentration of ACN (50%, 80%), TFA (0, 0.1%, 5%), acetic acid (0 or 0.1%), and/or glycolic acid (0, 0.1 M, 1 M) (Figure 3B). Optimal sulfopeptide recovery with Zr^4+^-IMAC was obtained in the presence of 80% ACN, 0.1% acetic acid (M10) (∼74 ± 20%, outperforming optimal TiO_2_ conditions). Notably, the relative recovery of phosphopeptides (∼39%) and the sY-unmodified peptide (∼17%) was also much reduced compared with optimal TiO_2_-enrichment conditions (Figure 2B), resulting in enrichment factors of either 3.55 or 1.88 when considering either all peptides or specificity with regard to phosphopeptide enrichment.

Increasing the acetonitrile concentration (from 50 to 80%) consistently increased the efficiency of sY-peptide recovery (cf. M7/M8, M9/M10, M4/M11, M5/M12). However, the type and concentration of acid had a greater effect on the efficiency of enrichment, with acetic acid being preferential for sY and TFA being preferential for pS/T-peptides (M7/M8, M9/M10 vs M4/M11, M5/M12). We also observed a reduction in sY enrichment factors with a decrease in glycolic acid concentration (from 1 to 0.1 M), primarily due to an increase in the recovery of unwanted phosphopeptides (compare M2/M3, M4/M5, M11/M12). That being said, the overall recovery of sY peptides was substantially reduced in the presence of any concentration of glycolic acid, negating the potentially positive effect on coenrichment of phosphopeptides (M4/M6, M1/M11, M7/M9, M8/M10) (Figure 3B).

### Challenges Associated with Accurate Site Localization of Sulfotyrosine within Tryptic Peptides

While there have been a number of studies exploring different strategies for sulfopeptide characterization and site localization (positive vs negative ion mode; fragmentation),^26,27,38,42,43,63,66,67,88,89^ the overall utility of these findings in terms of applicability for global discovery proteomics studies remains unknown; they either rely on instrumentation not commercially available or focus on a very small number of nontryptic peptides and are thus not representative of typical proteomics samples.

**Figure 4:**
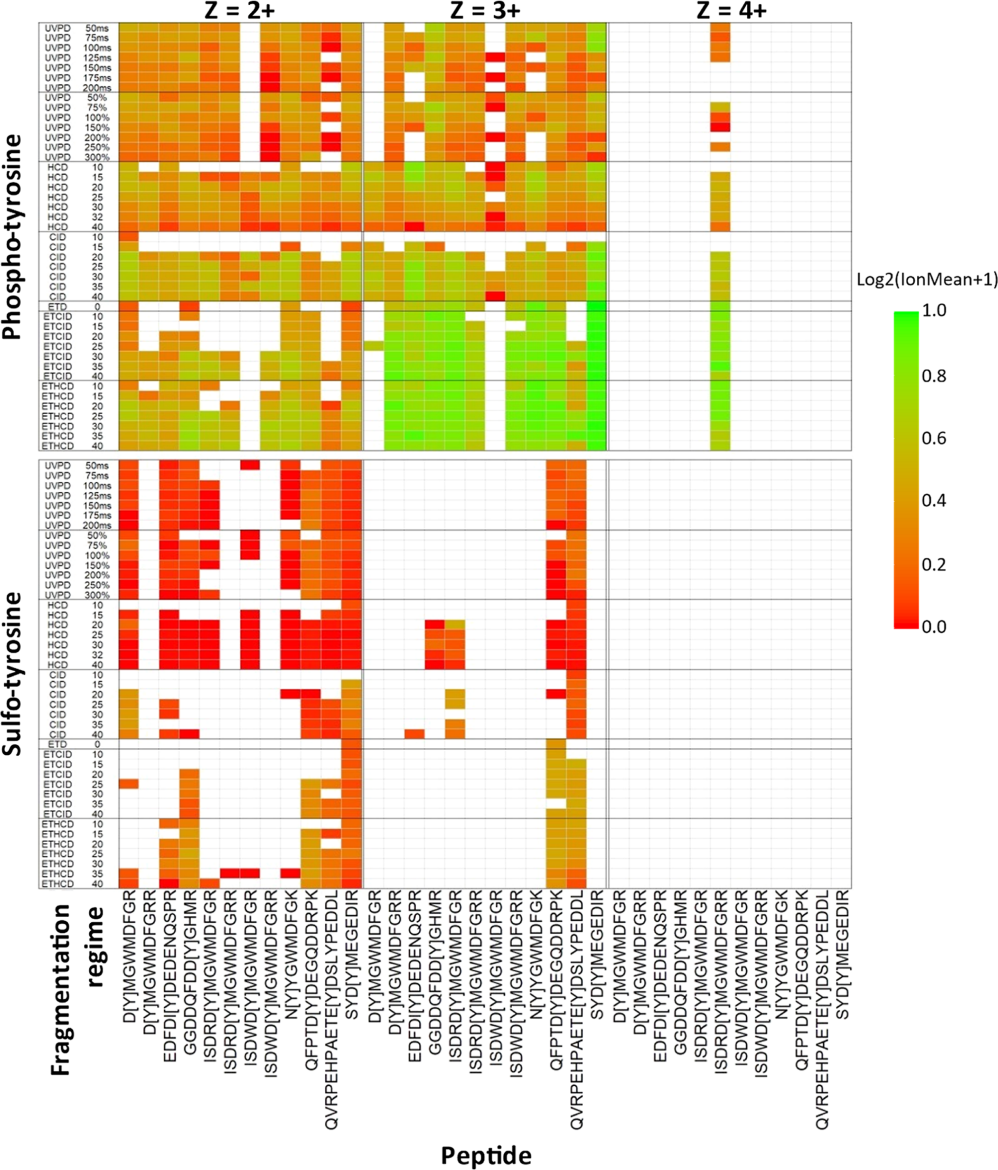
Comparison of site-determining ions for the sY and pY peptide panel using different fragmentation regimes. Two sample pools were generated containing either the panel of 12 sY- or 12 pY-containing peptides and subjected to LC–MS/MS on the Fusion Lumos Tribrid mass spectrometer (ThermoFisher) using fragmentation conditions as detailed. Ranging NCEs were applied for CID, HCD, ETciD, and EThcD as denoted. The ETD component was charge state-calibrated.^90^ UVPD activation time was either calibrated to molecular weight (%)^91^ or manually set (ms). Data were searched with COMET, and the highest scoring PSM was selected for further investigation. The heatmap shows the log_2_ ratio of the number of observed MS2 product ions containing the known modification mass shift (Δ80 Da) normalized to the total number of potential PTM-containing product ions (log_2_(ion mean + 1). Ions correlating to fragmentation at the same position in the peptide (e.g., *a*_4_/*b*_4_, *y*_8_/*y*_8_^2+^/*z*_8_) were collapsed into a single entry. The modification site is depicted by “[Y]”, and charge states are visualized separately. Green = all potential mass shift containing product ions detected; red = no potential mass shift containing product ions detected; white = no MS/MS spectra were confidently identified.

To better understand the ability to confidently localize sY sites in tryptic peptides (i.e., the generation of site-determining product ions), we characterized product ions generated from our synthetic panel of 12 sY tryptic peptides derived from known protein modifications using all potential fragmentation regimes available on a standard Fusion Lumos Tribrid instrument (ThermoFisher), namely, HCD, CID, ETD, EThcD, ETciD, and UVPD. We also evaluated 12 analogous pY peptides, allowing us to compare site localization confidence for both covalent modifications side-by-side. Considering the multiple settings available for each fragmentation regime, we investigated a total of 43 fragmentation conditions (Figure 4). sY and pY peptide libraries were analyzed by LC–MS/MS as separate pools and searched with COMET to aid analysis. For each fragmentation condition, peptide, and charge state, the number of observed product ions retaining the covalent modification was quantified as a function of the number of theoretically possible product ions for each (using the tandem mass spectrum with the highest COMET score; Figure 4). Unambiguous site localization ideally requires the generation of product ions that retain the covalent PTM. This is particularly important for sulfation, given the propensity for NL of Δ80 Da, resulting in ions that cannot be distinguished from those that would otherwise be unmodified. Data for ion types correlating with fragmentation around a particular residue (e.g., *a*_4_/*b*_4_/*y*_(*n*–4)_, *y*_8_/*y*_8_^2+^/*z*_8_) were compiled and treated as a single entity for the purpose of site localization.

As shown in Figure 4, there is stark disparity in the ability to localize the sites of modification on pY- versus sY-peptides under all of the conditions evaluated. Irrespective of the fragmentation strategy employed, and the energetics/time used, very few sulfonate-retaining product ions were observed for sY-containing peptides. ET(hc/ci)D yielded a higher proportion of sY- (and pY-) site determining ions than HCD for those peptides/conditions where product ions were observed, making electron-mediated fragmentation (EThcD at 25% NCE) optimal for localization of the sY sites in this peptide panel. However, even for these peptides, the relative proportion of site-determining ions was substantially lower than for the pY-peptide equivalents, and it is likely that the reduction in the sY-peptide ion charge state (Figures 1B and 4) prohibited efficient fragmentation (and thus identification) by EThcD/ETciD.^90,91^ In contrast with HCD, very few sulfopeptides were identified following CID. Where they were identified, there was almost complete NL of sulfonate, with the exceptions of D[Y]MGWMDFGR and SYD[Y]MEGEDIR. While HCD permitted sulfopeptide identification (for 9/12 peptides), the complete loss of 80 Da, even at low NCE (15%), meant that no site-determining ions were observed.

Our phosphopeptide data contrast significantly with that observed for the equivalent sulfopeptides, with varying degrees of phosphate retention seen for all peptides and charge states across all fragmentation strategies employed (Figure 4). For doubly protonated pY-peptide ions, overall phosphate retention was highest with CID_35, or EThcD_25, while the equivalent +3 ions benefitted substantially from ET(hc/ci)D, a finding well supported for phosphopeptides in previous studies.^41,57^ Both fragmentation regimes generally outperformed HCD in terms of the relative proportion of phosphopeptide-retaining product ions across all charge states. Interestingly, our observations with UVPD in terms of both automated peptide identification and generation of site-localizing ions broadly mirrored the results with HCD for both sY- and pY-peptides. However, UVPD was highly inefficient for peptide fragmentation, requiring long irradiation times (>100 ms) and generating product ions of low intensity, irrespective of peptide sequence, charge, or modification status. sY-peptides were generally well identified with UVPD (8 out of 12 sY-peptides). However, sulfonate loss was extensive, and it was not possible to localize the precise sites of sulfation on our sY-peptide panel.

### sY- and pY-Peptides Can Be Discriminated Based on Precursor NL with HCD at Low Energy (10% NCE)

A number of strategies have been introduced to help distinguish sY from pY in peptides and proteins. These include a subtractive approach based on phosphatase treatment and acetylation of free (unmodified) tyrosine residues.^66^ However, subtractive analytic approaches fundamentally rely on both complete phosphate removal and subsequent chemical modification of unmodified tyrosine residues, where inefficiencies in either step will result in mis-identification. The presence of additional labile tyrosine PTMs (such as nitration) also increases the potential of sY mis-identifications. Adduction of sY with trace metal ions (from LC solvents and ESI emitters) has also been reported to improve sY peptide identification and site localization.^63^ As well as increasing the average charge state (thus increasing ETD/EThcD efficiency), metal-adducted sY-peptides have been reported to be more likely to retain the sulfate moiety (permitting localization by ETD mediated fragmentation regimes). However, while the relative proportion of Na^+^/K^+^ adducts of the 2 sY-peptides reported in a previous study was relatively high, we failed to observe any sY-peptide metal-ion adduction following manual interrogation and open PTM searching of our data. Unfortunately, the addition of Na^+^/K^+^ salts into LC–MS systems compromises instrument performance and is thus not a practical solution.

**Figure 5:**
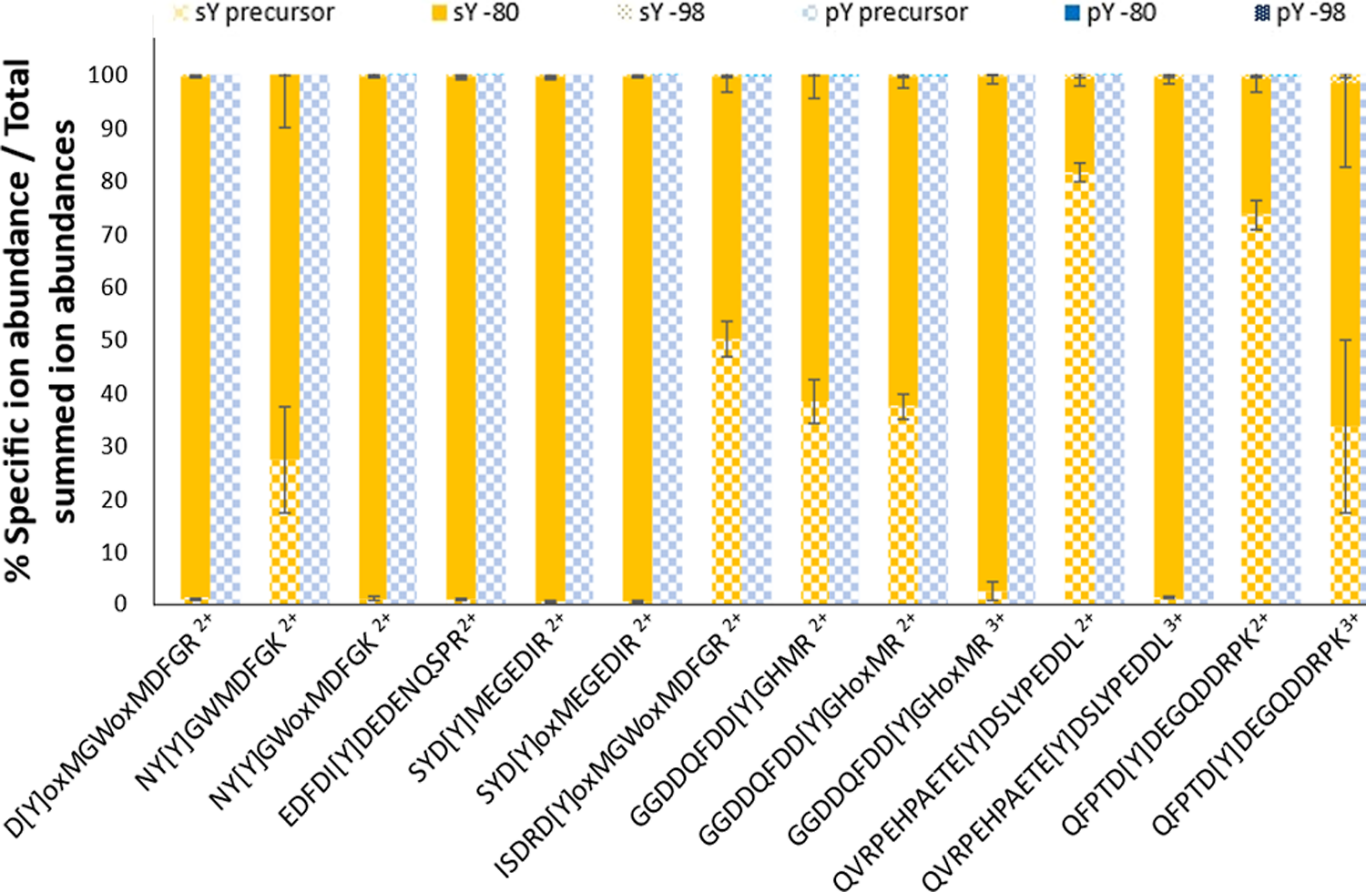
NL propensity of sY- vs pY-peptides at 10% NCE HCD fragmentation. The most intense PSM (determined from the .mgf file) for each sY and pY peptide at different charges (including oxidized methionine variants) were selected for quantitation (14 peptide ions). The relative abundance (%) of the precursor ion (check), precursor ion −80 Da (block), or precursor ion −98 Da (dotted) was calculated as a proportion of the summed intensity of all ions in the MS2 spectrum. sY-containing peptides in yellow; pY peptides in blue.

Our observations (Figure 4), and that of others,^27,92^ reveals a marked difference in the propensity of PTM NL in both HCD and CID between sY- and pY-peptides. We thus hypothesized that we could exploit this feature to develop a low-energy NL HCD triggering approach that could discriminate near-isobaric PTMs and enable sY-peptide identification from complex mixtures containing pY peptides. To test this, we quantified HCD precursor ion NL at 10% NCE for sY- and pY-peptides, quantifying −80 Da (SO_3_/HPO_3_) and −98 Da (H_2_SO_4_/H_3_PO_4_) mass shifts (Figure 5 and S2). For each peptide spectrum match (Figure 4), the relative abundance of the precursor or NL precursor ions was calculated as a percentage of total product ion current (Figure 5). The predominant 10% NCE HCD product ion observed across our sY-peptide panel equated to loss of 80 Da (sulfonate) from the precursor, accounting for 20–100% of the MS/MS ion current (median = ∼85%). Little to no peptide backbone cleavage was observed, in agreement with the finding that no peptides were identified with the search engine under this condition (Figure 4). In contrast, the pY-peptide exhibited minimal loss of either 80 or 98 Da (<1%) under the same conditions (Figure 5 and S2).

Interestingly, while the degree of NL for sY peptide ions appeared to be dictated by the ratio between the number of basic residues and the charge state, the trend was the opposite to that observed for phosphopeptides.^93^ Where the charge state was greater than the number of basic residues (H/K/R), contributing to a “mobile proton environment”, we observed near complete NL of 80 Da. However, the degree of precursor ion NL was comparatively reduced when the charge state was smaller than the number of basic sites (−80 Da NL: QFPTD[Y]DEGQDDRPK: +2 ∼20%, +3 ∼65% and QVRPEHPAETE[Y]DSLYPEDDL: +2 ∼15%, +3 ∼95%). These data suggest that the charge-directed fragmentation mechanisms that appear to drive phosphopeptide NL^93,94^ are not directly applicable to sulfopeptides, whose fragmentation (propensity for NL) is also likely to be affected by differences in the electronegativity and hydrogen bonding capabilities of the sulfonate moiety.

Having demonstrated the ability to distinguish sY- and pY-peptides based on the HCD precursor ion NL at 10% NCE, we next sought to implement this for sY-peptide identification using an NL triggering approach in a mixture. Given optimal peptide identification across all charge states with HCD (32% NCE) (Figure 4), we used the 80 Da precursor ion loss at 10% NCE HCD to trigger 32% NCE HCD on the same precursor to permit peptide identification, analyzing a 1:1 ratio of our sY- and pY-peptide panel. Using this approach, we were able to positively identify all of the sY-peptides and did not identify any of the pY-containing peptides, confirming the utility of this low energy HCD triggering strategy. To our knowledge, this is the first reported case of utilizing low collision energy HCD fragmentation to efficiently distinguish sY- and pY-peptides by MS in a single run.

### Application of Our Sulfopeptide Analytical Pipeline to Identify the Secreted “Sulfome” of HEK-293 Cells

**Figure 6:**
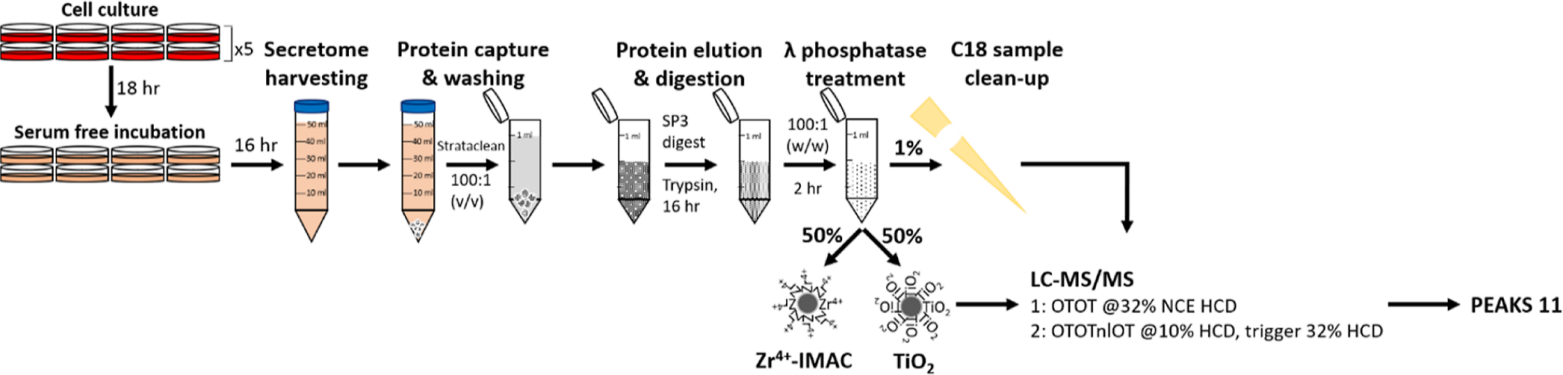
Workflow for the identification of sulfated peptides from a HEK-293 cell secretome. Following incubation in serum-free media, the secretome was harvested, and proteins were captured with Strataclean beads. Eluted proteins were subject to SP3-based trypsin proteolysis and treatment with λ phosphatase. 1% was subjected to C18 sample cleanup prior to LC–MS/MS analysis. The remainder was subject to enrichment using either of the two optimized protocols employing Zr^4+^-IMAC or TiO_2_. All samples were analyzed using (i) high resolution (Orbitrap, OT) DDA with 32% NCE HCD or (ii) NL triggering strategy where loss of 80 Da at 10% NCE HCD invoked precursor fragmentation with 32% NCE HCD. Data were analyzed using PEAKS 11.

sY-proteins are generated in the Golgi compartment and are predominantly destined for secretion or cell membrane localization: of the 33 validated human sY-proteins in UniProt, 19 are secreted and 14 are membrane-bound.^6^ In order to test the capabilities of our optimized workflow for sTyr, we evaluated the secretome of the adherent HEK-293 model cell system (Figure 6). After an 18 h incubation in serum-free medium, the HEK-293 cell secretome was collected, purified, and prepared using Strataclean resin and an SP3-based trypsin digestion protocol (adapted from ref (95)) and treated with protein phosphatase.

Given the differences in pY and sY peptide enrichment between Zr^4+^-IMAC and TiO_2_ protocols, we elected to compare both optimized enrichment protocols to investigate the cellular secretome. However, initial analysis of enriched material from agarose coated (Purecube) Zr^4+^-IMAC resin using a standard data-dependent acquisition (DDA) pipeline revealed extensive binding of nonmodified peptides. We thus employed MagReSyn Zr-IMAC HP (ReSyn Biosciences), which exhibits substantially reduced nonspecific peptide binding.^96^

**Figure 7:**
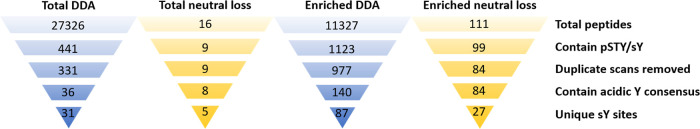
Evaluation of sulfopeptide enrichment and NL triggering data acquisition for sulfopeptide identification. Secretome samples were analyzed using either a DDA strategy (32% NCE HCD) or the 10% NCE HCD NL triggered strategy for HCD acquisition (32% NCE), with or without enrichment using TiO_2_/Zr^4+^-IMAC enrichment (Figure 6). Listed are the total number of peptides identified, those containing either pSTY or sY before or after the removal of duplicate scan PSMs. Peptide lists were subsequently filtered for those where the site of modifications was in an acidic consensus (with D/E at the +1 or −1 position relative to Y) and then for unique sY sites. Numbers are representative of ≥2 independent experiments.

To allow us to evaluate (i) the efficiency of sY- versus pY-peptide enrichment using the TiO_2_ and MagReSyn Zr^4+^-IMAC HP resins and (ii) the NL triggered-strategy for sulfopeptide identification, we acquired MS/MS data using a DDA pipeline alongside our NL-triggered approach (Figures 6 and 7).

To confirm the effective purification of the secretome, 1% of the sample was subject to DDA analysis using HCD (NCE 32%). Performing an initial open PTM search (PEAKs PTM), we identified a high degree of Met oxidation (∼5900 PSMs) and Asn/Gln deamidation (∼5500 PSMs). Met oxidation and Asn deamidation as well as Ser/Thr/Tyr phosphorylation and Tyr sulfation were thus included as variable modifications in subsequent searches. From the total secretome DDA analysis, we identified 27,326 peptides from 2,695 proteins at a 1% FDR (Figure 7). Gene Ontology (GO) analysis with DAVID (Table S2) revealed that 36% of the identified proteins were defined as being localized to extracellular exosomes (Benjamini–Hochberg corrected *p*-value <1.6 × 10^–163^), 29% as membrane-bound (Benjamini–Hochberg corrected *p*-value = 6.49 × 10^–56^) and 44% as cytosolic (Benjamini–Hochberg corrected *p*-value = 7.72 × 10^–101^). Of these ∼27k peptides, 441 (<2%) were annotated as containing either pS/T/Y or sY (330 and 141 sites, respectively) (Figure 7). A substantive proportion (∼25%) of these annotations were from duplicate scan numbers where the same scan generated PSMs annotated as containing either pSTY or sY or there were differences in the deamidation status of Asn/Gln. Manually filtering this list and retaining the highest scoring scan number unique PSMs yielded 331 identifications. Enzymatic deposition by TPST1/2 is known to occur on Tyr residues within an acidic consensus (with Asp or Glu localized at either the +1 or −1 position). Therefore, we further filtered these identifications based on an acidic consensus, revealing 36 peptides, 31 of which were potential unique sites of Tyr sulfation (Figure 7).

A total of 11,327 peptides were identified following DDA analysis of both enriched samples. No substantive differences in terms of isoelectric point or *m*/*z* distribution were observed for those PSMs identified from the total or the enriched secretome samples (Figure S3). Ten percent of the identifications from the enriched samples (1,123) were annotated as phosphorylated and/or sulfated (898 and 304, respectively). While markedly lower than the level of enrichment typically seen from a cell extract using standard phosphopeptide enrichment strategies^57,97−99^ (usually ∼85–90% using TiO_2_ in our hands), this sulfopeptide enrichment strategy yielded over a 6-fold increase in the identification of modified peptides from the secretome sample and a ∼2.2-fold increase in the peptides annotated as being sulfated. Removal of duplicate scans (977 identifications) and filtering for an acidic consensus left some 140 peptides with 87 potential sY sites (Figure 7).

Performing our NL-triggered MS acquisition method with the same sample significantly reduced the number of peptides identified in both the total (0.06%) and enriched (1.0%) samples. The numbers of peptides identified in the enriched DDA experiment compared to the enriched NL analysis indicate that the enrichment strategy alone is insufficient for enhanced sulfopeptide identification (in agreement with our synthetic peptide analysis, Figure 3) but that enrichment does serve to improve the sensitivity of the MS acquisition.

Of the two different enrichment strategies, the sample prepared using Zr^4+^-IMAC HP resin triggered the greatest number of MS/MS spectra (demonstrating higher likely enrichment of sY-peptides) following 10% NCE HCD, yielding 977 MS2 spectra; the TiO_2_-enriched sample resulted in 754 triggering events. Despite >1,700 total triggering events, only 327 spectra were matched to a peptide sequence (∼19%), suggesting issues associated with fragmentation and/or ion intensity that compromised identification. Surprisingly, of these 327 PSMs, 319 (∼98%) were from the TiO_2_ sample, with only 8 (∼2%) spectra from the Zr^4+^-IMAC HP resin. We are currently at a loss to explain this marked difference in the ratio of triggering events to peptide identifications. While there was a slight decrease in the precursor ion intensity and *m*/*z* of Zr^4+^-IMAC-enriched peptides compared with those from the TiO_2_ sample, these were not substantive, and the overall distributions of *m*/*z*, mass, charge and ion intensity were comparable (Figure S4). In terms of total peptide identifications, this equates to 111 for TiO_2_ of which 62 were annotated by the software as sY-containing (54% enrichment efficiency), and 7 for Zr^4+^-IMAC of which 6 were annotated as sulfated (86% enrichment efficiency). Applying the acidic-Tyr filter and concatenating for the highest scoring PSM per scan for our enriched NL triggered data set (Figure 7), we identified 84 modified peptides, all of which contained a Tyr residue within an acidic motif. The resultant 27 unique sites of modification mapped to 23 proteins (Tables 1 and S3).

**Table 1:**
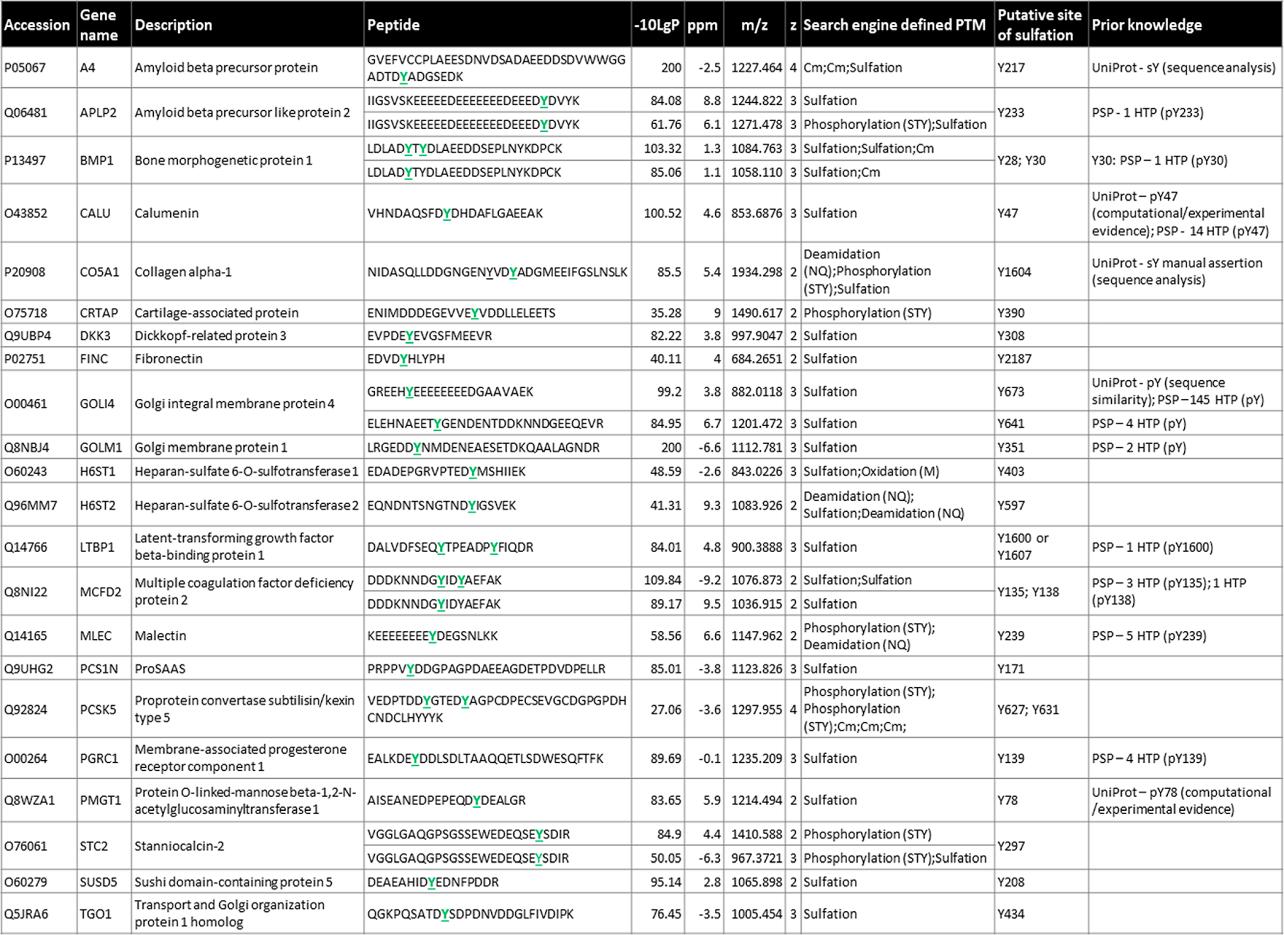
Confidently Identified sY-Peptides from the HEK293 Cellular Secretome^a^

a Detailed are the accession number, gene name, protein description, peptide sequence, −log_10_*P* score, Δppm from theoretical *m*/*z*, the observed *m*/*z*, charge state (*z*), and identified PTMs as determined by the search engine (1% FDR). Listed is a concatenated version of all modified peptide identifications (all peptides having been identified in at least two separate experiments, with multiple PSMs per experiment), maintaining only the highest −log_10_*P* score peptide for a unique sY-site. The predicted (acidic) sY-site is highlighted. Cm—carbamidomethylation. All predicted sY sites were searched for prior annotation as being either phosphorylated or sulfated in UniProt and the PhosphositePlus (PSP) database [accessed July 2023]; HTP—details of the number of times of phosphorylation were reported in PSP in a high throughput study.

Importantly, while the numbers of “identified” sites of sulfation were greater with the DDA experiments than following our NL-triggered approach, manual interrogation of these data revealed that, in the vast majority of cases, these appear to be mis-identified phosphopeptides based on preferential loss of 98 rather than 80 amu. Of the 27 sulfated peptides that were identified from the enriched NL analysis, only 20 were observed with DDA; 77% of the 87 identifications were thus deemed to be incorrectly assigned by the search algorithm. Likewise, for the nonenriched sample, while all 5 sulfopeptides seen following NL-triggering were observed in the DDA experiment, the other 26 appear to be mis-identified phosphopeptides. Additionally, our enriched NL-triggering method was able to identify 7 additional sites of sulfation that were not observed by any other approach, showing that this strategy is both more efficient and provides greater confidence in the identification of sulfated peptides.

Considering that our NL triggering MS acquisition method can efficiently discriminate sY- from pY peptides, we attempted to utilize confidence scores (−log_10_*P*) and Δppm to distinguish between pSTY/sY values of these duplicated scans (Figure S5). The majority of PSMs were annotated as being sulfated, with or without Asn/Gln deamidation. Although Δppm was generally lower for those PSMs with a higher −log_10_*P* value, this was not consistent. PSMs annotated as deamidated generally had both a higher Δppm and were lower scoring. We did not observe any score or ppm-related features that could confidently differentiate assigned PSMs as being either phosphorylated or sulfated. We thus concatenated the data, removing the lower scoring duplicate PSM to retain a single identification per scan; this yielded a final data set of 23 different peptides and 30 pSTY/sY annotations from the HEK-293 secretome (Table 1).

We next compared our data set of 27 modified peptides with the human Tyr “Sulfome” in UniProt. Interestingly, only Y217 on the amyloid β precursor protein (A4, P05067) and Y1604 on collagen α-1(V) (CO5A1, P20908) have been previously identified as sites of sulfation. While other sites of sY have been reported in both of these proteins, these were not identified in our data. A number of these, including Y262 on A4, are situated in extremely acidic regions, which likely do not lend themselves to trypsin-based identification.

Six of the 27 peptides contain multiple sites of (+80 Da) modification, being annotated as doubly sulfated, doubly phosphorylated, or a mixture of the two. Focusing, in the first instance, on a doubly “phosphorylated” peptide from PCSK5 (proprotein convertase subtilisin/kexin type 5), two of the five Tyr residues have an Asp at −1 and are localized within a highly acidic region (the others being clustered at the peptide C-terminus). It is also worth noting that the tandem MS data that generated these identifications were triggered based on NL of 80 Da at 10% NCE HCD, which we have shown does not induce phosphate loss (Figure 5 and S2). Like the mixed PTM containing peptides (sY and pS/T/Y) identified from APLP2 (amyloid β precursor like protein 2), CO5A1 (collagen α-1), and STC2 (stanniocalcin-2), it is therefore likely that (at least) one of these sites is in fact sulfated; we assume that NL from the sulfated residue under lower energy conditions triggered MS/MS data acquisition and identification of a peptide that is also phosphorylated. Likewise, the singly modified site on STC2 (which is embedded in an EYxD motif) is likely also sulfated. To investigate this further, we interrogated the 10% HCD scans for these precursors, determining that the MS2 spectra from both APLP2 and STC2 contained an NL peak equivalent to a single sulfated residue (−80 Da), while spectra for the secreted metalloproteinase BMP1, MCFD2, CO5A1, and PCSK5 contain losses equivalent to (80 and) 160 Da, suggestive of two sites of tyrosine sulfation. This agrees with information in UniProt that identifies Y1601 on CO5A1 as an additional site of sulfation, strongly indicative of mis-identification of sulfation sites as phosphosites by the search engine.

Given our synthetic peptide panel data (Figures 1B, 4, and S1), an unexpected finding from our final secretome-derived (nonunique) data set was the large number of ions (61 out of the 84 peptides, i.e., >60%) that were observed with a charge state ≥3. The sulfation-induced reduction in charge state observed for the panel meant that we had expected a substantive proportion of those peptides observed using our sulfoproteome pipeline (Figure 6) to be doubly protonated. In fact, while this was true across all peptides in the DDA data set from the enriched secretome, this was not the case for the NL-triggered data set, with the majority of peptide ions appearing as +3 species (Figure S6A). We believe that this is because the peptides identified with the NL triggering approach are much larger (and thus of higher mass) compared to those from the enriched DDA set (Figure S6B,C) or our peptide panel; this can be explained by virtue of the fact that the sY-containing (NL-triggering) peptides from the secretome contained a substantially higher proportion of acidic residues (9.0 D/E residues on average) compared with those peptides in the total protein DDA set (average of 1.7 D/E residues) (Figure S6D), and consequently a lower relative proportion of K/R residues.

To validate the secretome data obtained using our pipeline for identification of Tyr sulfation, we expressed and affinity-precipitated the novel sulfated substrates H6ST1 and H6ST2 (Golgi-localized Heparan-sulfate 6-*O*-sulfotransferase 1/2) from HEK-293T cells and subjected both proteins to *in vitro* PAPS-dependent sulfation with recombinant TPST1/2. We also immunoprecipitated the related isoform H6ST3, which contains an analogous Tyr residue within the acid consensus sequence in its C-terminal region. After enzymatic reaction, proteins were digested with trypsin and analyzed by LC–MS/MS using both DDA and our NL-triggered method. As well as confirming TPST1/2-dependent sulfation of H6ST1 and H6ST2 at the same sites on tryptic peptides observed from our global discovery study (sTyr403 and sTyr597, respectively, Tables S4 and S5), we also show that the related protein, H6ST3, can also be Tyr sulfated at sTyr285 and sTyr464, (Tables S4 and S5). Of particular interest, sTyr403, sTyr597, and sTyr 464 lie in a conserved acidic motif in the C-terminal region of H6ST1-3 outside of the catalytic domain (Figure S7). Not only does this analysis validate the exploratory potential of our discovery pipeline for sTyr detection, but it also demonstrates how this information can be extrapolated to predict additional sites of modification in closely related proteins such as H6ST3. Interestingly, only one of the two TPST1/2-dependent sulfation sites on HS6ST3 contained an acidic residue in close proximity (sTyr464-[ED**Y**X]), suggesting that an acidic motif around the site of sulfation may not be an absolute requirement for TSPT1/2 substrates.

As well as validating our sulfomics pipeline, our enzymatic assays also revealed additional novel TPST1/2-dependent sulfation sites on proteins that coimmunoprecipitated with H6ST2 and/or H6ST3: Gem-associated protein 5 (GEMIN5; sTyr992), tubulin α-1A/B/C chain (TUBA1A/TUBA1B/TUBA1C; sTyr161, sTyr432), tubulin β chain (TUBB; sTyr[50 or 51], sTyr340), insulin receptor substrate 4 (IRS4; sTyr921), and heterogeneous nuclear ribonucleoprotein H (HNRNPH1; sTyr266). Gemin5 is thought to reside in the nucleoplasm and in specific nuclear bodies (Gemini of Cajal Bodies) as well as the cytoplasm and has not previously been shown to be sulfated (or modified on Tyr992). Likewise, none of the other TSPT1/2 substrates has previously been reported to be sulfated. However, the *in vitro* sulfated Tyr sites identified on these proteins have previously been reported as being Tyr phosphorylated in PhosphoSitePlus in HTP proteomics screens, with IRS4 Tyr921 described as a putative substrate for the Tyr protein kinases Fer and IGFR1. Finally, we also identified what we believe to be the first auto-Tyr sulfation site on TPST1 (likely at Tyr326), which might itself be relevant to cellular TPST1/2 regulation or enzyme/substrate interactions, similar to the signaling paradigm in which tyrosine kinases are themselves controlled by tyrosine phosphorylation.

Together, these data provide strong evidence that our discovery sulfomics pipeline is capable of defining novel human sulfopeptides and suggest Tyr sulfation cross-talk as a potential regulatory modification between Golgi colocalized human heparan sulfate sulfotransferases and TPSTs.

## Conclusions

In this study, we investigated several strategies for the analytical discrimination of sulfopeptides from phosphopeptides by employing, to our knowledge, the largest panel of synthetic peptides based on tryptic human peptides derived from known sites of sY-modifications from cellular proteins. In developing a workflow specifically for “sulfomics”, we characterized a number of sY-peptide discriminatory features that include RT shifts, susceptibility (or lack thereof) to treatment with protein phosphatase, conditions for sY-peptide enrichment, and peptide fragmentation. We developed and implemented the first discovery “sulfomics” pipeline and demonstrated its utility using a HEK-293 cellular secretome, where we identified a total of 21 novel (experimentally determined) sY-containing proteins and 28 sY-sites, which expands the known human “sulfome” by ∼70%. In the process of validating H6ST1 and H6ST2 (and HS6ST3) as *in vitro* targets of the sulfotransferases TSPT1/2, we also identified additional *in vitro* substrates—GEMIN5, TUBA1, TUBB, IRS4 and HNRNPH1—which are themselves part of the known H6ST2/3 interactome. Interestingly, while the site on GEMIN5 has not previously been identified as being modified, there is extensive HTP evidence of the phosphorylation of the other TSPT1/2 substrates. It thus remains to be seen whether (i) these sites can be both sulfated and phosphorylated *in vivo*, (ii) our identification of these specific sulfation sites *in vitro* is biased by the assay conditions, or (iii) the phosphorylation site identifications reported in PhosphoSite Plus are actually mis-representations of a Tyr sulfation event. Since Tyr sulfation is believed to be irreversible, our findings raise the possibility that false identification of sTyr as pTyr may be meaningful for several proteins and urgently needs to be clarified for multiple proteins. In addition, since sTyr could act as a Golgi-based signal that competes with, or prevents, Tyr phosphorylation further along the secretory pathway, we are in the process of evaluating this combinatorial phenomenon. Regardless, the identification of both glycan and protein sulfotransferases as sY-containing proteins potentially opens up a new research area for understanding the regulation and substrate targeting of these enzymes, similar in many ways to the phosphoregulatory paradigms established for protein kinases.^100^

While Fe^3+^/Ga^3+^ IMAC has previously been used for sY peptide enrichment,^25,26^ we were unable to validate these findings using our peptide library. However, we demonstrate the utility of Zr^4+^-IMAC and TiO_2_ in acetic acid-based solutions for the semispecific enrichment of sY-peptides in a complex (phospho) peptide mixture. Noting the differential enrichment of the relatively acidic nonmodified peptides from our library (average pI ∼ 4.5), compared with unmodified peptides from BSA/casein (average pI ∼ 5.7), our evidence suggests that the acidic consensus (thought to be required for TPST1/2 directed sulfation) also contributes to the efficiency of sY-peptide enrichment. This is also reflected in the complex secretome analysis, where the NL-triggered enriched peptides (sY-containing) identified were of much greater acidity than the total secretome DDA identifications (pI ∼ 4.6 vs ∼6.2).

In undertaking comprehensive MS analysis of tryptic sulfopeptides using CID, HCD, EThcD, ETciD and UVPD fragmentation regimes, we were able to quantify the generation of site-localizing product ions and compare them with equivalent synthetic phosphopeptides. Taken together, our data indicate that specific sulfosite localization is poor, irrespective of fragmentation regime or conditions used. Our data with the commercially available UVPD configuration did not prove as promising as anticipated, based on previous work.^38,42,43^ A number of factors likely contribute to this: difference in UVPD wavelength, energy, and/or laser frequency, the use of positive rather than negative ion mode, and the fact that many of the studies reported to date use a handful of exemplar peptides. EThcD appeared to be the best overall for site localization but was inconsistent in its ability to identify sulfopeptides, in part because of the reduction in charge state. During the course of our studies, we determined that low energy (10% NCE) HCD could serve to differentiate sulfopeptides from phosphopeptides, based on the lability of the sulfate group. We thus made use of the single feature that caused the greatest analytical challenge in terms of localization to allow sY discrimination “on the fly”; a low energy (10% NCE) HCD NL-triggering approach to define sulfopeptide-containing scans for subsequent identification. Our initial thinking based on the reduced ionization efficiency of sulfopeptides was that application of EThcD to global “sulfome” analysis would be compromised. However, our secretome-derived data set suggests that the average increase in length and charge state of sulfopeptides may make an NL-triggered EThcD strategy feasible for sulfosite identification. However, this strategy will need to be evaluated for throughput given the additional time requirements of EThcD over HCD and the necessity given that (i) most peptides only contain a single Tyr residue and (ii) the strong requirements for an acidic consensus for TSPT1/2 activity.

Thus, while the pipeline described herein facilitates the identification of sY-peptides from complex mixtures, unambiguously pinpointing the precise site of Tyr modification remains challenging. Specifically, the electronegativity of sY and issues associated with site localization in positive ion mode MS (given current fragmentation strategies) continue to compromise definitive site determination unless peptides only contain a single Tyr residue.

Recent reports have commented on the inherent metal ion-binding affinity of sY that might be exploited for localization,^27,92,101^ with one report identifying metal adducted species as dominant when using standard positive ion mode LC–MS/MS methods (specifically Na^+^/K^+^,^63^). As such, we performed open PTM searches on our data sets in an attempt to identify some of the missing triggered peptides (86% of triggers). However, we failed to observe these species in our investigations. No additional sY-containing proteins were identified with the open PTM search, although differentially modified species of already identified sY peptides were observed (i.e., containing either Met ox or Asn deamidation). We presume that this is a direct result of how PEAKs PTM performs its searches, requiring a protein to be identified by a first round database search with predefined search parameters, prior to performing a second round mass shift search on a concatenated database containing only these identified proteins.^102^ Since our NL triggering method drastically reduces the number of peptides and proteins identified, this compromises the ability for PEAKS PTM to identify additional sulfated peptides in the absence of defining specific additional modifications. In summary, we believe that the analytical developments reported in this paper and the optimized pipeline for discovery sulfome analysis, incorporating phosphatase treatment, an optimized TiO_2_-based enrichment protocol, NL-triggered HCD MS/MS acquisition, and appropriate data analysis considerations, provide a resource to the community to better define the extent and roles of protein sulfation from biological samples.

## Supporting Information Available

Figure S1. Charge state distribution of our standard panel of peptides in an unmodified, phosphorylated, or sulfated state. Figure S2. Sulfated peptides but not phosphopeptides yield extensive precursor ion loss of 80 amu following 10% NCE HCD fragmentation. Figure S3. Isoelectric point versus *m*/*z* distribution of the peptides identified from either the total DDA unenriched HEK-293 secretome sample, or following enrichment with TiO_2_ or Zr^4+^-IMAC. Figure S4. Characteristics of precursor ions that triggered 32% NCE HCD following precursor ion NL at 10% NCE HCD following secretome peptide enrichment with either BioResyn Zr^4+^-IMAC (BRzr) or TiO_2_. Figure S5. Distinguishing duplicated scan PSMs. Figure S6. Properties of peptides from enriched HEK293 secretome. Figure S7. Sequence conservation of a biochemically/MS-validated sulfated Tyr residue that lies C-terminal to an acidic motif in the C-terminus of human (h) Heparan Sulfate 6OST1, 2 and 3 (PDF)
Table S1. Synthesized peptide standards (XLSX)
Table S2. Gene ontology analysis of DDA total protein identifications (XLSX)
Table S3. HEK293 secretome sulfopeptide identification and supporting data (XLSX)
Table S4. Proteins and peptides identified following *in vitro* sulfation of immunoprecipitated heparan sulfate 6-*O*-sulfotransferases (H6STs) with TPST1/2 (XLSX)
Table S5. Proteins and sulfopeptides identified following in vitro sulfation of immunoprecipitated H6ST1, H6ST2 or H6ST3 with TPST1/2 (XLSX)

## Notes

All MS data has been deposited at the ProteomeXchange Consortium (http://proteomecentral.proteomexchange.org) via the PRIDE partner repository with the data set identifiers PXD043713 and PXD043723.
