# Supplementary material for: Custom Workflow for the Confident Identification of Sulfotyrosine-Containing Peptides and Their Discrimination from Phosphopeptides
Source: J Proteome Res. 2023 Nov 8;22(12):3754–72. doi: 10.1021/acs.jproteome.3c00425 (PMC10696596; doi:10.1021/acs.jproteome.3c00425)
Supplement: Supplementary file 1 — pr3c00425_si_001.pdf [file pr3c00425_si_001.pdf]

Supporting Information

Custom workflow for the confident identification of sulfotyrosine-containing peptides and their discrimination from phosphopeptides

Leonard A. Daly[1,2], Dominic P. Byrne[2], Simon Perkins[3], Philip J Brownridge[1], Euan McDonnell[2, 3] Andrew R. Jones[2, 3], Patrick A. Eyers[2], and Claire E. Eyers[1,2]*.

[1] Centre for Proteome Research, Institute of Systems, Molecular & Integrative Biology, University of Liverpool, Crown Street, Liverpool, L69 7ZB, U.K.

[2] Department of Biochemistry, Cell & Systems Biology, Institute of Systems, Molecular & Integrative Biology, University of Liverpool, Crown Street, Liverpool L69 7ZB, U.K.

[3] Computational Biology Facility, Institute of Systems, Molecular & Integrative Biology, University of Liverpool, Crown Street, Liverpool, L69 7ZB, U.K.

*Corresponding author: Claire E. Eyers (ceyers@liverpool.ac.uk).

Table of Contents

Figure S1. Charge state distribution of our standard panel of peptides in an unmodified, phosphorylated, or sulfated state. (PDF)

Figure S2. Sulfated peptides but not phosphopeptides yield extensive precursor ion loss of 80 amu following 10% NCE HCD fragmentation. (PDF)

Figure S3. Isoelectric point versus *m/z* distribution of the peptides identified from either the total DDA unenriched HEK-293 secretome sample, or following enrichment with TiO_2 or Zr^{4+}-IMAC. (PDF)

Figure S4. Characteristics of precursor ions that triggered 32% NCE HCD following precursor ion NL at 10% NCE HCD following secretome peptide enrichment with either BioResyn Zr4+-IMAC (BRzr) or TiO_2. (PDF)

Figure S5. Distinguishing duplicated scan PSMs. (PDF)

Figure S6. Properties of peptides from enriched HEK293 secretome. (PDF)

Figure S7. Sequence conservation of a biochemically/MS-validated sulfated Tyr residue that lies C-terminal to an acidic motif in the C-terminus of human (h) Heparan Sulfate 6OST1, 2 and 3. (PDF)

Table S1. Synthesized peptide standards. (XLSX)

Table S2. Gene ontology analysis of DDA total protein identifications. (XLSX)

Table S3. HEK293 secretome sulfopeptide identification and supporting data. (XLSX)

Table S4. Proteins and peptides identified following in vitro sulfation of immunoprecipitated Heparan Sulfate 6-O-Sulfotransferases (H6STs) with TPST1/2. (XLSX)

Table S5. Proteins and sulfopeptides identified following i*n vitro* sulfation of immunoprecipitated H6ST1, H6ST2 or H6ST3 with TPST1/2. (PDF)

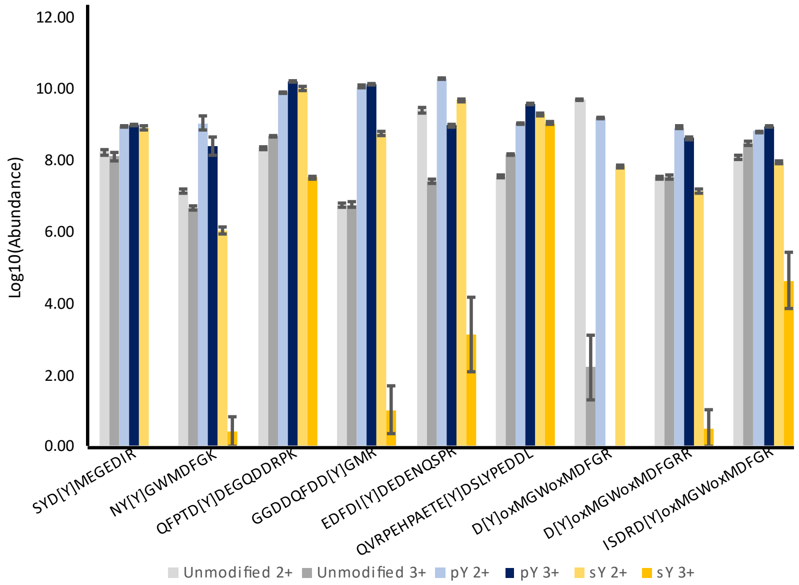

Figure S1. Charge state distribution of our standard panel of peptides in an unmodified, phosphorylated, or sulfated state. The most intense PSM (determined from the mgf file) for each sY and pY peptide at different charges (including oxidised methionine variants) were selected for quantitation (14 peptide ions). The absolute abundance (log10) of the precursor ion (check), precursor ion -80 Da (block), or precursor ion -98 Da (dotted) is presented. sY-containing peptides in yellow; pY peptides in blue. Modification site denoted by [Y], ox = oxidized residue. Error bars represent SE from N = 10 replicates.

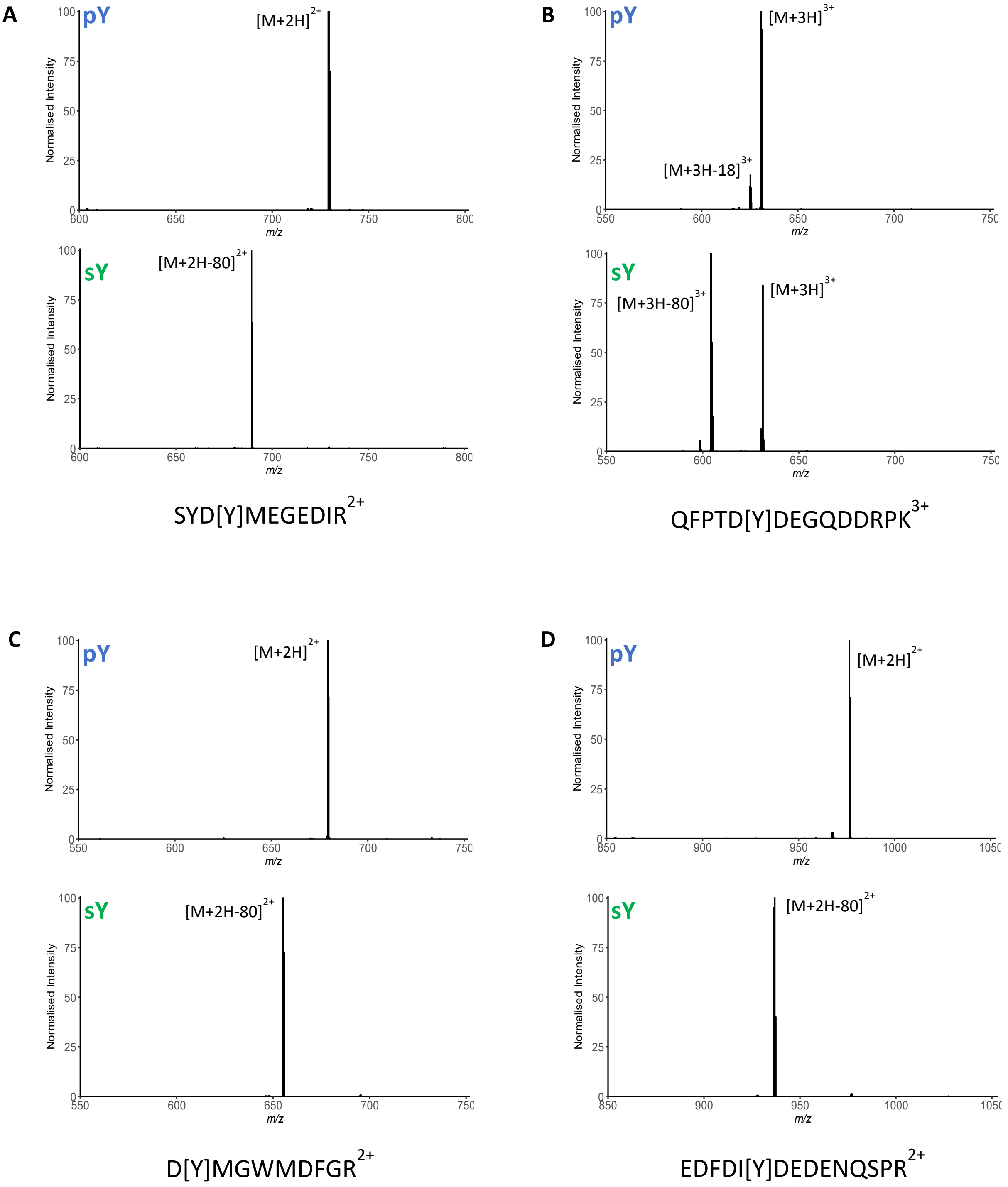

Figure S2. Sulfated peptides but not phosphopeptides yield extensive precursor ion loss of 80 amu following 10% NCE HCD fragmentation. MS2 spectra of identical peptide sequences containing either sY (bottom) or pY (top) were subjected to HCD fragmentation using 10% NCE. A) doubly protonated SYD[Y]MEGEDIR ion at *m/z* 729.3; B) triply protonated QFPTD[Y]DEGQDDRPK ion at *m/z* 630.9; C) doubly protonated D[Y]MGWMDFGR ion at *m/z* 679.2; D) doubly protonated EDFDI[Y]DEDENQSPR ion at *m/z* 976.4. Spectra were redrawn using a custom R script from the .mgf file and major peaks annotated.

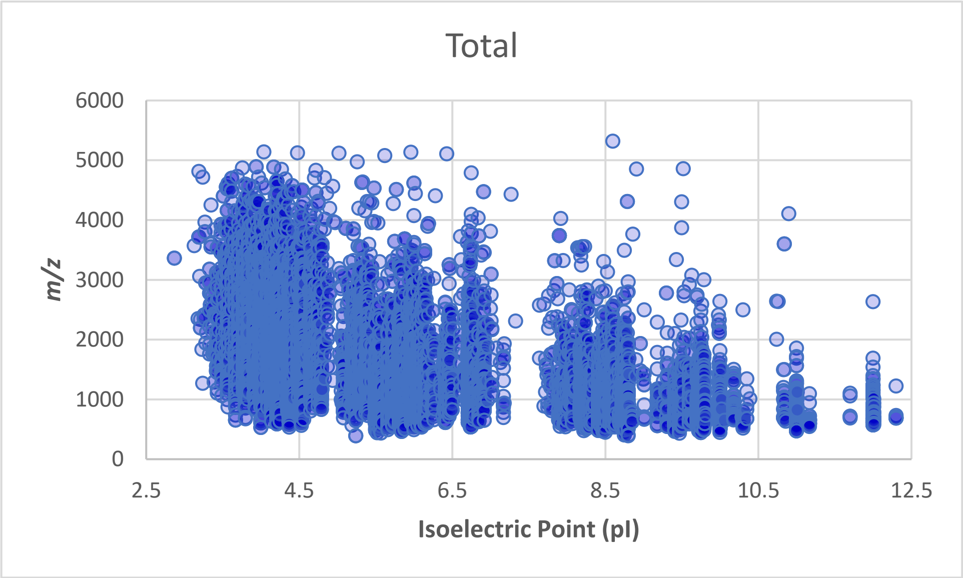

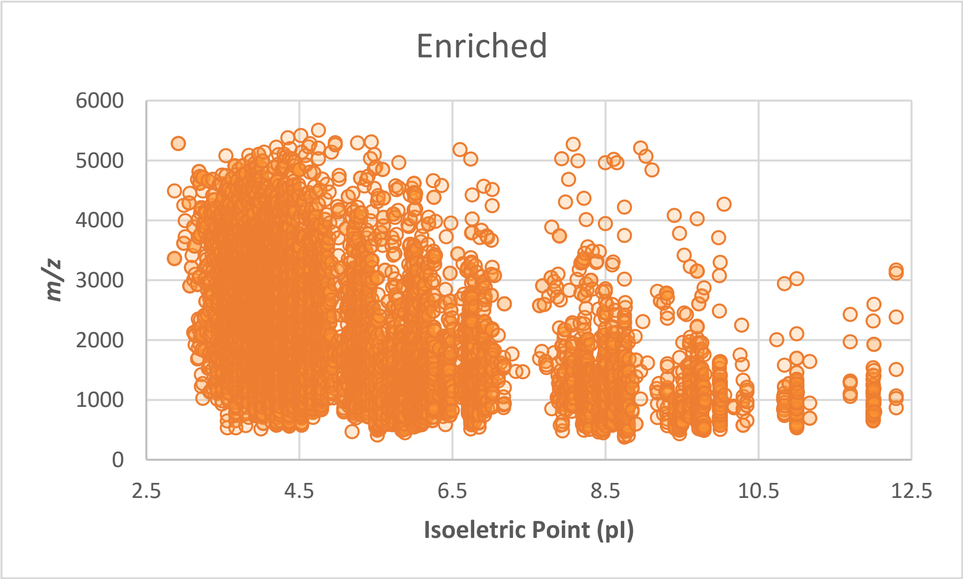

Figure S3. Isoelectric point versus *m/z* distribution of the peptides identified from either the total DDA unenriched HEK-293 secretome sample (top; blue), or following enrichment with TiO_2 or Zr^{4+}-IMAC (bottom; orange). *m/z* data was extracted from PEAKs11 analysis. Identified peptide sequences were input into the Expasy compute_pI tool to determine pI (https://web.expasy.org/compute_pi/) [accessed June 2023].

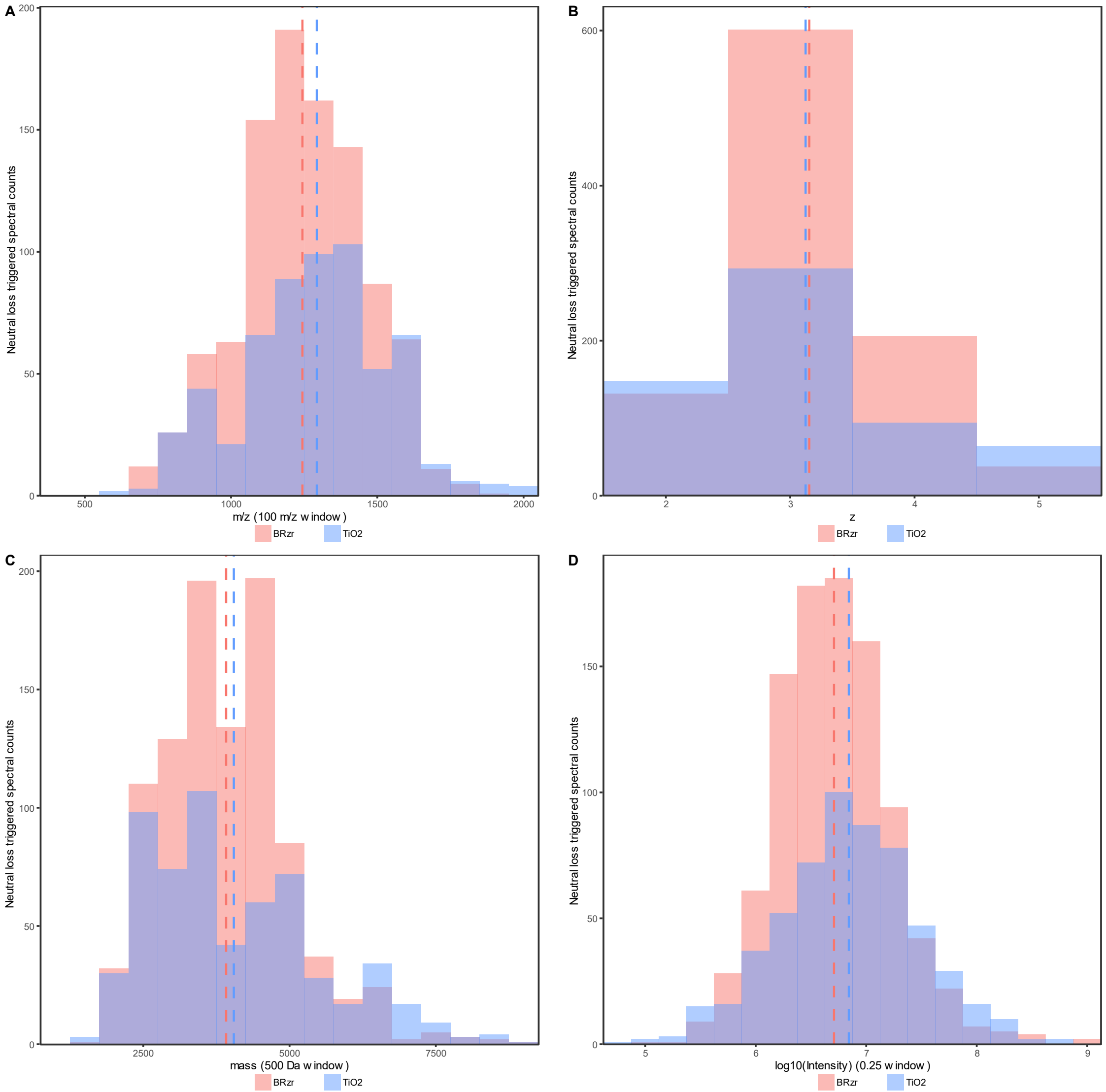

Figure S4. Characteristics of precursor ions that triggered 32% NCE HCD following precursor ion NL at 10% NCE HCD following secretome peptide enrichment with either BioResyn Zr4+-IMAC (BRzr) or TiO_2. The number of neutral loss triggered spectral counts is plotted for the two enriched samples (BioResyn Zr^{4+}-IMAC HP (red) or TiO_2 (blue)) as a function of: A) Precursor ion *m/z* in 100 *m/z* windows. B) Precursor ion charge state. C) Precursor mass in 500 Da windows. D) Precursor log10(intensity) in 0.25 windows. Data were extracted from .mgf files where the MSconvert "HCD energy = 32" filter was set to specifically identify triggered scans.

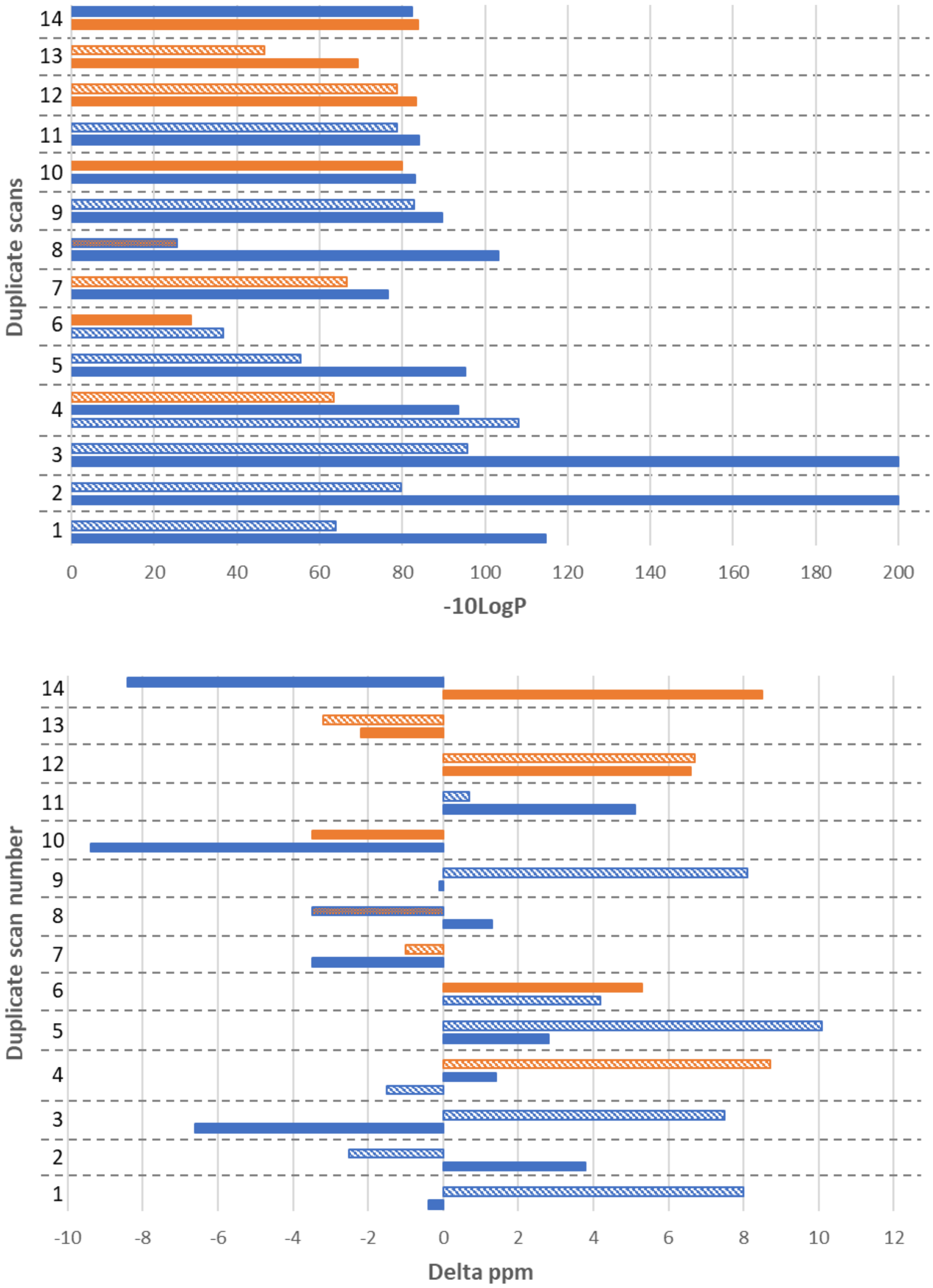

Figure S5. Distinguishing duplicated scan PSMs. -10LogP value (top) and Δppm (bottom) for 14 representative duplicate scans from the neutral loss triggered enriched dataset with different PTMs assigned to the same peptide sequence. Sulfation - blue; phosphorylation – orange; deamidation – check boxes. Better score often, but does not always, correlate with a lower Δppm.

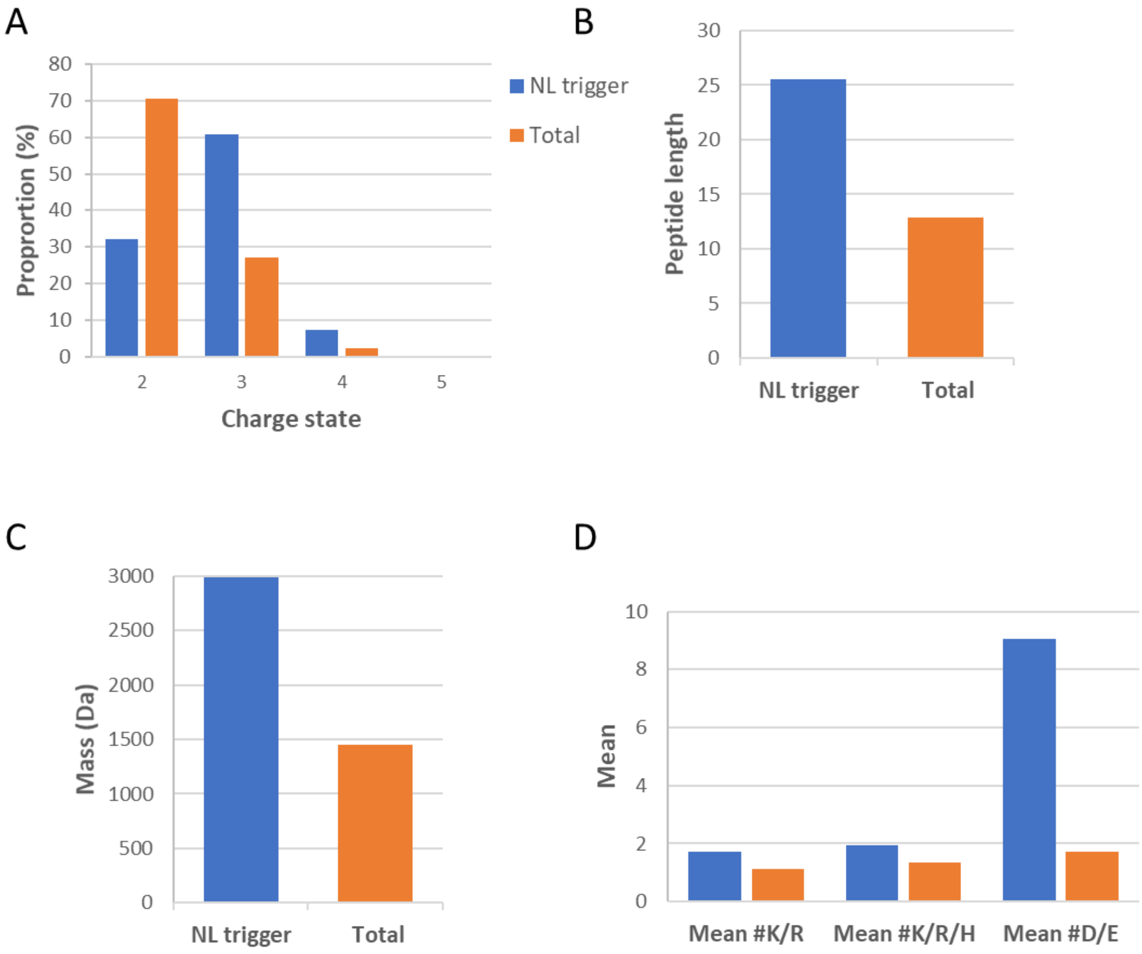

Figure S6. Properties of peptides from enriched HEK293 secretome. All non-duplicated PSMs from either the neutral loss triggered enriched (blue) or the total unenriched protein DDA (orange) analyses were interrogated for (A) charge state; (B) peptide length; (C) average mass; (D) average (mean) number of K/R/H or D/E residues per peptide. All data was extracted from the PEAKs11 output.

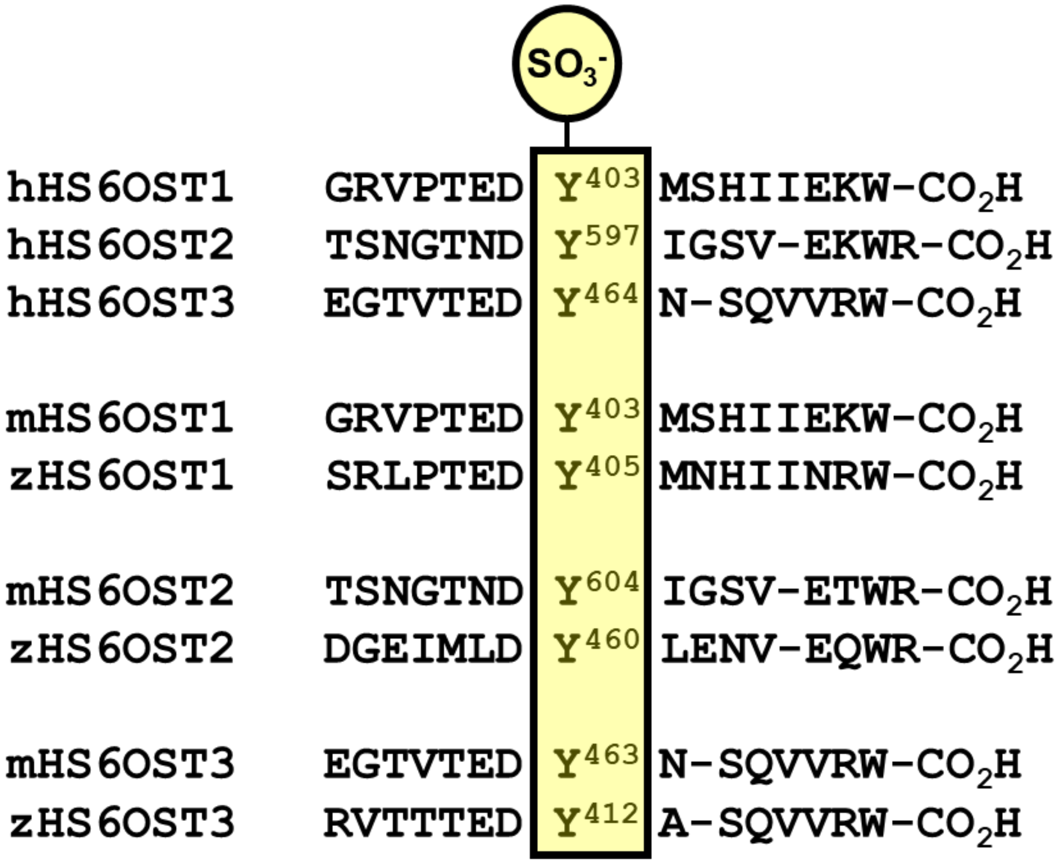

Figure S7. Sequence conservation (shaded) of a biochemically/MS-validated sulfated Tyr residue (Table S5) that lies C-terminal to an acidic motif in the C-terminus of human (h) Heparan Sulfate 6OST1, 2 and 3. The aligned sequence of the equivalent C-terminal region from mouse (m) and zebrafish (z) is also shown.

Condition	Gene name	Protein name	% Coverage	Mascot Score	Sulfopeptide Sequence	Region in protein	Ion Score
H6ST1 no TPST1/2 (DDA)	HS6ST1	Heparan-sulfate 6-O-sulfotransferase 1	52	1530			
	HS6T3	Heparan-sulfate 6-O-sulfotransferase 3	22	548			
H6ST1 +TSPST1/2 (DDA)	HS6ST1	Heparan-sulfate 6-O-sulfotransferase 1	59	1599	EDADEPGRVPTED**Y**MSHIIEK	390-410	44
					EDADEPGRVPTED**Y**MSHIIEKW	390-411	28
	HS6T3	Heparan-sulfate 6-O-sulfotransferase 3	9	381			
H6ST1 + TPST1/2 (NL)	HS6ST1	Heparan-sulfate 6-O-sulfotransferase 1	7	120	EDADEPGRVPTED**Y**MSHIIEK	390-410	59
					EDADEPGRVPTED**Y**MSHIIEKW	390-411	51
					EDADEPGRVPTED**Y**MSHIIEKW	390-411	27
	TPST1	Protein-tyrosine sulfotransferase 1	9	61	LG**Y**DP**Y**ANPPNYGKPDPK	324-341	52
H6ST2 no TPST1/2 (DDA)	HS6ST2	Heparan-sulfate 6-O-sulfotransferase 2	30	627			
	HS6ST3	Heparan-sulfate 6-O-sulfotransferase 3	19	197			
H6ST2 + TPST1/2 (DDA)	HS6ST2	Heparan-sulfate 6-O-sulfotransferase 2	43	1395	EQNDNTSNGTND**Y**IGSVEK	585-603	95
	GEMIN5	Gem-associated protein 5	1	32	V**Y**EAVELLK	991-999	32
	TPST1	Protein-tyrosine sulfotransferase 1	53	2821	LG**Y**DPYANPPNYGKPDPK	324-341	48
H6ST2 + TPST1/2 (NL)	HS6ST2	Heparan-sulfate 6-O-sulfotransferase 2	3	105	EQNDNTSNGTND**Y**IGSVEK	585-603	106
	TPST1	Protein-tyrosine sulfotransferase 1	5	83	LG**Y**DP**Y**ANPPNYGKPDPK	324-341	42
	TUBA1A	Tubulin alpha-1A chain	6	48	EDMAALEKD**Y**EEVGVDSVEGEGEEEGEEY	423-451	48
H6ST3 + TPST1/2 (DDA)	HS6ST3	Heparan-sulfate 6-O-sulfotransferase 3	63	2453	SPTPDELPTC**Y**PGDDWSGVSLR	275-296	42
					EDGAAEGTVTED**Y**NSQVVR	452-470	59
					DHQWPKEDGAAEGTVTED**Y**NSQVVR	446-470	71
	TPST2	Protein-tyrosine sulfotransferase 2	60	3709	VLKGD**Y**K	348-354	33
	TUBA1B	Tubulin alpha-1B chain	65	1713	LSVD**Y**GKK	157-164	30
					EDMAALEKD**Y**EEVGVDSVEGEGEEEGEEY	423-451	44
	TUBA1C	Tubulin alpha-1C chain	60	1249	LSVD**Y**GKK	157-164	30
	HNRNPH1	Heterogeneous nuclear ribonucleoprotein H	22	225	DLN**Y**CFSGMSDHR	263-275	31
H6ST3 + TPST1/2 (NL)	HS6ST3	Heparan-sulfate 6-O-sulfotransferase 3	10	475	DHQWPKEDGAAEGTVTED**Y**NSQVVR	446-470	104
					EDGAAEGTVTED**Y**NSQVVR	452-470	77
					SPTPDELPTC**Y**PGDDWSGVSLR	275-296	43
	IRS4	Insulin receptor substrate 4	1	128	EADSSSD**Y**VNMDFTK	914-928	49
					EADSSSD**Y**VNMDFTKR	914-929	97
	TUBB	Tubulin beta chain	8	108	ISV**YY**NEATGGK	47-58	65
					NSS**Y**FVEWIPNNVK	337-350	59
	TUBA1A	Tubulin alpha-1A chain	8	69	EDMAALEKD**Y**EEVGVDSVEGEGEEEGEEY	423-451	50
					LSVD**Y**GKK	157-164	39
	TPST1	Protein-tyrosine sulfotransferase 1	5	55	LG**Y**DP**Y**ANPPNYGKPDPK	324-341	47
	HNRNPH1	Heterogeneous nuclear ribonucleoprotein H	3	43	DLN**Y**CFSGMSDHR	263-275	43

Table S5. Proteins and sulfopeptides identified following in vitro sulfation of immunoprecipitated H6ST1, H6ST2 or H6ST3 with TPST1/2. H6ST1, H6ST2 or H6ST3 were overexpressed in HEK-293 cells, immunoprecipitated and subjected to *in vitro* sulfation with TPST1/2. Samples were digested with trypsin and subjected LC-MS/MS analysis, using either data-dependent acquisition (DDA) or the low energy (10% NCD HCD) neutral loss (NL) triggering strategy. Detailed are the conditions for the assay, identified proteins of interest, protein sequence coverage (%) and Mascot score, sulfopeptide sequence, region in the protein and peptide score. Putative site of sulfation in the peptide sequence is underlined. No modified peptides were observed in the absence of TPST1/2.
